# Extended treatment duration and recurrence in successfully treated drug-susceptible pulmonary tuberculosis with cavity on chest radiograph

**DOI:** 10.1371/journal.pone.0357772

**Published:** 2026-09-10

**Authors:** Chang-Seok Yoon, Tae-Ok Kim, Hong-Joon Shin, Ju Sang Kim, Hyung Woo Kim, Eung Gu Lee, Sung Soo Jung, Jee Youn Oh, Jin Woo Kim, Heayon Lee, Seunghoon Kim, Sun-Hyung Kim, Yeonhee Park, Jiwon Lyu, Sun Jung Kwon, Yun-Jeong Jeong, Do Jin Kim, Hyeon-Kyoung Koo, Ganghee Chae, Sun Young Kyung, Jinsoo Min, Yong-Soo Kwon

**Affiliations:** 1 Department of Internal Medicine, Chonnam National University Hospital, Chonnam National University Medical School, Gwangju, Republic of Korea; 2 Division of Pulmonary and Critical Care Medicine, Department of Internal Medicine, Incheon St. Mary’s Hospital, College of Medicine, The Catholic University of Korea, Incheon, Republic of Korea; 3 Division of Pulmonary, Allergy, and Critical Care Medicine, Department of Internal Medicine, Bucheon St. Mary’s Hospital, College of Medicine, The Catholic University of Korea, Bucheon, Republic of Korea; 4 Division of Pulmonary and Critical Care Medicine, Department of Internal Medicine, Chungnam National University Hospital, Daejeon, Republic of Korea; 5 Division of Pulmonary, Allergy, and Critical Care Medicine, Department of Internal Medicine, Korea University Guro Hospital, Korea University College of Medicine, Seoul, Republic of Korea; 6 Division of Pulmonary and Critical Care Medicine, Department of Internal Medicine, Uijeongbu St. Mary’s Hospital, College of Medicine, The Catholic University of Korea, Uijeongbu, Republic of Korea; 7 Division of Pulmonary, Critical Care, and Sleep Medicine, Department of Internal Medicine, Eunpyeong St. Mary’s Hospital, College of Medicine, The Catholic University of Korea, Seoul, Republic of Korea; 8 Division of Pulmonary and Critical Care Medicine, Department of Internal Medicine, St. Vincent’s Hospital, College of Medicine, The Catholic University of Korea, Suwon, Republic of Korea; 9 Division of Pulmonary and Critical Care Medicine, Department of Internal Medicine, Chungbuk National University Hospital, Cheongju, Republic of Korea; 10 Division of Pulmonary and Critical Care Medicine, Department of Internal Medicine, Daejeon St. Mary’s Hospital, College of Medicine, The Catholic University of Korea, Daejeon, Republic of Korea; 11 Department of Pulmonary and Critical Care Medicine, Soonchunhyang University Cheonan Hospital, Soonchunhyang University College of Medicine, Cheonan, Republic of Korea; 12 Division of Pulmonary and Critical Care Medicine, Department of Internal Medicine, Konyang University Hospital, Konyang University College of Medicine, Daejeon, Republic of Korea; 13 Division of Pulmonary and Critical Care Medicine, Department of Internal Medicine, Dongguk University Ilsan Hospital, Goyang, Republic of Korea; 14 Division of Allergy and Respiratory Medicine, Soonchunhyang University Bucheon Hospital, Soonchunhyang University College of Medicine, Bucheon, Republic of Korea; 15 Division of Pulmonary and Critical Care Medicine, Department of Internal Medicine, Ilsan Paik Hospital, Inje University College of Medicine, Goyang, Republic of Korea; 16 Division of Pulmonary and Critical Care Medicine, Department of Internal Medicine, Ulsan University Hospital, Ulsan University College of Medicine, Ulsan, Republic of Korea; 17 Division of Pulmonology, Department of Internal Medicine, Gachon University Gil Medical Center, Incheon, Republic of Korea; 18 Division of Pulmonary and Critical Care Medicine, Department of Internal Medicine, Seoul St. Mary’s Hospital, College of Medicine, The Catholic University of Korea, Seoul, Republic of Korea; Ajou University School of Medicine, KOREA, REPUBLIC OF

## Abstract

**Background:**

Cavity on chest radiograph is recognized as an independent risk factor for recurrence in pulmonary tuberculosis (TB), but evidence supporting extended treatment in these patients remains limited, particularly in real-world data from Korea.

**Methods:**

We performed a retrospective analysis using data from a multicenter prospective cohort of patients with pulmonary tuberculosis enrolled at 18 Korean institutions, comparing recurrence rates between standard treatment (≤200 days) and extended treatment (>200 days) among drug-susceptible pulmonary TB patients with cavity on chest radiograph.

**Results:**

Among 159 patients, 58 (36.5%) received standard treatment and 101 (63.5%) received extended treatment. Baseline sputum smear positivity was higher in the extended group (29.3% vs. 56.4%, p = 0.002), but 2-month culture positivity (6.3% vs. 4.4%, p > 0.999) and time to culture conversion (8.00 ± 3.91 vs. 7.45 ± 3.73 weeks, p = 0.454) were similar. Recurrence during post-treatment follow-up occurred in 4 patients (2.5%) and did not differ between groups (3.4% vs. 2.0%, p = 0.966). Treatment interruption due to adverse drug reactions was more frequent with extended treatment (1.7% vs. 7.9%, p = 0.204), while overall adverse event rates were comparable. In a fixed-time analysis using Firth’s penalized logistic regression, extended treatment showed a numerically lower risk of recurrence, although statistical significance was not reached (adjusted odds ratio [aOR], 0.07; 95% confidence interval [CI], 0.01–1.06; p = 0.056). Among covariates, low body mass index was associated with recurrence, but this estimate was very imprecise and should be interpreted with caution.

**Conclusion:**

Cavity on chest radiograph alone may not be sufficient to justify routine extension of treatment beyond 200 days. Because only four recurrence events were observed, the adjusted association favoring extended treatment was imprecise and should be interpreted as exploratory rather than definitive. Larger prospective studies that systematically capture microbiologic response and detailed radiologic disease burden are needed to identify which patients with cavitary disease may benefit from individualized treatment extension.

## Introduction

Pulmonary tuberculosis (TB) is an infectious disease caused by *Mycobacterium tuberculosis* and transmitted via inhalation of airborne droplet nuclei generated by patients with infectious pulmonary TB. It is a preventable and treatable disease, with treatment success rates approach 95% in drug-susceptible (DS) TB when managed with the standard 6-month regimen [1,2]. However, advanced TB can lead to significant lung destruction and the development of cavitary lesions. The prevalence of cavitary TB has been reported to range from 29% to 87% [3].

Cavitary lesions in the lungs are associated with severe disease, including higher bacterial loads in sputum, and have been linked to an increased risk of post-treatment recurrence [4–10]. Recurrence is clinically important because it is associated with morbidity and mortality and may contribute to the development of drug-resistant TB [1].

To reduce recurrence risk in higher-risk phenotypes, the 2016 guidelines from the American Thoracic Society (ATS), Centers for Disease Control and Prevention (CDC), and Infectious Diseases Society of America (IDSA) recommend extending the continuation phase by 3 months, based largely on historical trial evidence and expert opinion, for patients with cavity on chest radiograph, particularly when cultures remain positive at 2 months [8]. However, contemporary evidence supporting routine treatment extension in cavitary TB remains limited.

Treatment extension increases cumulative drug exposure and may increase the risk of adverse drug reactions, treatment interruptions, and additional costs. Drug resistance in *M. tuberculosis* arises through selection of spontaneous mutations under drug pressure, and inadequate or interrupted therapy can facilitate acquired resistance [11]. Therefore, optimizing treatment duration requires balancing potential benefits against the burden of longer therapy. We aimed to evaluate whether extending treatment beyond 200 days is associated with a reduced risk of bacteriologically confirmed recurrence during the post-treatment follow-up period among patients with DS pulmonary TB who had cavity on baseline chest radiograph.

## Methods

### Population

**Fig 1:**
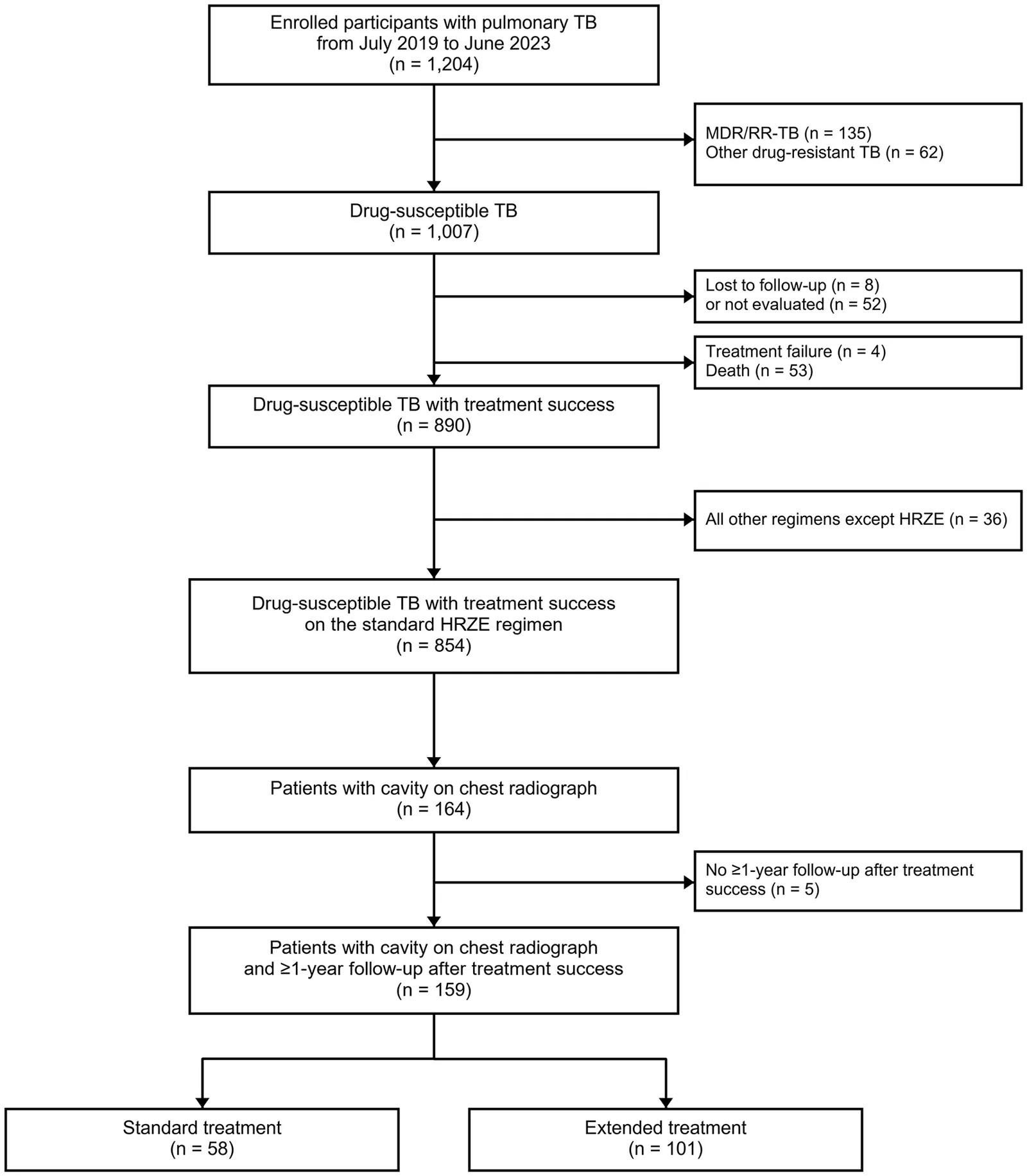
Flow chart of participant enrollment. Abbreviations: TB: tuberculosis; MDR/RR: multi-drug resistance/rifampin-resistance; HRZE: H: isoniazid; R: rifampin; Z: pyrazinamide; E: ethambutol.

A prospective cohort of 1,204 pulmonary TB patients who were recruited from 18 centers in South Korea between July 2019 and June 2023 was analyzed. Details of the cohort protocol have been described previously [12]. The data for this study were accessed on 30 June 2024. To evaluate the efficacy and safety of extended treatment in patients at high risk of recurrence, those with cavity on chest radiograph were included. Patients with any evidence or history of drug-resistant TB were excluded, including multidrug-resistant or rifampin-resistant TB, isoniazid mono-resistant TB, and other forms of mono-resistant TB. Accordingly, the analytic population was restricted to patients with pan-susceptible TB, defined as susceptibility to all standard HRZE components. Among these patients, only those who received the standard isoniazid, rifampin, pyrazinamide, and ethambutol (HRZE) regimen and achieved treatment success were included. Therefore, patients who did not receive the HRZE regimen, experienced treatment failure, died, transferred to other clinics, were lost to follow-up, or discontinued treatment for reasons such as consent withdrawal or changes in diagnosis were excluded. The final analytic cohort consisted of patients with cavity on chest radiograph who completed post-treatment follow-up (Fig 1).

### Data collection

For the selected patients, baseline demographic data such as age, sex, and comorbidities were recorded at the time of diagnosis. Comorbidities were defined based on documented diagnoses, relevant medication use, or available clinical information in the medical records. Diabetes mellitus and hypertension were defined by documented diagnoses or use of glucose-lowering or antihypertensive medications, respectively. Chronic pulmonary disease included asthma, chronic bronchitis, emphysema, or other chronic pulmonary diseases with ongoing respiratory symptoms. Chronic kidney disease and chronic liver disease were defined as documented chronic renal or hepatic disease. Cardiovascular disease included chronic cardiac conditions such as ischemic heart disease, heart failure, or arrhythmia; neurologic disease included chronic neurologic disorders such as stroke, dementia, or Parkinson’s disease; and malignancy included active or previous cancer. Additionally, initial clinical data, including vital signs, biochemical tests, and microbiological results, were documented. Biochemical tests, including complete blood count, liver function tests, renal function tests, and inflammation markers, were conducted through blood sampling. To evaluate cavity or multilobe infiltration, chest radiographs were assessed, and for microbiological evaluation, acid-fast bacilli (AFB) smear and culture were evaluated through sputum tests.

Throughout the follow-up period, participants received regular biochemical evaluations and were closely monitored for adverse drug reactions at predefined intervals (14 days, 28 days, 2 months, and monthly thereafter). Adverse drug reactions were identified from the cohort medical records and categorized using operational definitions adapted from CTCAE version 5.0 where applicable. Although information on clinical severity was available in some medical records, formal CTCAE severity grades were not uniformly recorded in the cohort database. Therefore, adverse drug reactions were analyzed as binary outcomes based on their presence or absence rather than by severity grade. All patients were followed for a minimum of one year after treatment completion through regular outpatient visits, linkage with the national tuberculosis surveillance system, and telephone follow-up when necessary to ensure complete ascertainment of recurrence [12–14].

### Group and study outcome

The enrolled patients were categorized according to total treatment duration. Because the standard 6-month regimen corresponds to approximately 180 days, extended treatment was defined as treatment lasting >200 days, allowing an additional 2 weeks beyond the expected standard duration. This 2-week allowance was based on a previous study of prolonged TB treatment [15], which considered that treatment interruptions of ≥2 weeks during the intensive phase may require reintroduction of intensive-phase treatment and thereby prolong total treatment duration. Thus, the 200-day cutoff was selected to distinguish meaningful treatment extension from minor variations around the standard 6-month course.

The primary outcome was recurrence, defined as bacteriologically confirmed tuberculosis occurring within the follow-up period after treatment completion in patients previously classified as cured or having successfully completed treatment.

### Statistical analysis

Continuous variables are presented as means ± standard deviations, and categorical variables are presented as counts (%). When comparing the two groups, t-tests were used to compare the means of continuous variables, while Pearson’s chi-square test or Fisher’s exact test were used to analyze the associations between categorical variables.

To identify factors associated with recurrence, logistic regression analyses were conducted. Because extended treatment was defined as a total treatment duration of >200 days, a fixed-time analysis at 200 days was used to ensure consistent exposure classification. Cox proportional hazards modeling was not used as the main analytic approach because total treatment duration was determined during treatment and was fully known only after treatment completion; therefore, treating it as a baseline exposure could introduce immortal-time bias. Time-dependent Cox modeling or modeling treatment duration as a continuous variable was also not applied as the main analytic approach because only four recurrence events were observed, making more complex models highly unstable. Given the small number of recurrence events and the presence of separation, both univariable and multivariable analyses were conducted using Firth’s penalized logistic regression. Univariable analyses were first performed using demographic characteristics, comorbidities, radiologic findings, and microbiologic results. Given the very small number of recurrence events, we sought to minimize overfitting by restricting the number of variables included in the multivariable model. Extended treatment was included as the main exposure of interest. Age and sex were included a priori as clinically important covariates, and variables with p < 0.05 in the univariable analysis were additionally considered for inclusion.

All statistical analyses were performed using R software (version 4.5.1; R Foundation for Statistical Computing, Vienna, Austria).

### Ethics statement

The study was conducted in accordance with the Declaration of Helsinki and was approved by the Institutional Review Boards (IRBs) and Ethics Committees of all participating institutions. The study was coordinated and overseen by the representative institution, the Catholic University of Korea (IRB No. KC19ONDI0458), which represented and managed the study across the 17 other participating medical institutions. All participating institutions obtained IRB approval prior to study initiation, with the following approval numbers: SCHBC202012023001 (Soonchunhyang University Bucheon Hospital), UUH202103016 (Ulsan University Hospital), GCIRB2021−016 (Gachon University Gil Hospital), 2020GR0402 (Korea University Guro Hospital), VC19ONDI0168 (St. Vincent's Hospital), KANGDONG2020-06-012-001 (Kangdong Sacred Heart Hospital), KYUH2020-12-013-002 (Konyang University Hospital), SCHCA202012076 (Soonchunhyang University Cheonan Hospital), DUIH202101023001 (Dongguk University Ilsan Hospital), 2020-07-007 (Chungnam National University Hospital), 2020-08-012-003 (Chungbuk National University Hospital), DC19ONDI0052 (Daejeon St. Mary's Hospital), HC19ONDI0068 (Bucheon St. Mary's Hospital), UC19ONDI0106 (Uijeongbu St. Mary's Hospital), PC19ONDI0149 (Eunpyeong St. Mary's Hospital), OC19ONDI0073 (Incheon St. Mary's Hospital), and ISPAIK2020-11-003-001 (Ilsan Paik Hospital). Written informed consent was obtained from all adult participants before their enrollment in the study.

## Results

Of the 1,204 patients in the cohort, 135 with multidrug-resistant or rifampin-resistant TB, 49 with isoniazid mono-resistant TB, and 13 with other mono-resistant TB were excluded. Additional exclusions included patients who did not receive the HRZE regimen (n = 36), experienced treatment failure (n = 4), died (n = 53), transferred to other clinics (n = 41), were lost to follow-up (n = 8), or discontinued treatment because of consent withdrawal or changes in diagnosis (n = 11). Consequently, 854 pan-susceptible DS TB patients who achieved treatment success with the standard HRZE regimen were identified; 164 had cavity on chest radiograph, and 159 completed follow-up after treatment completion and were included in the final analytic cohort (Fig 1).

A total of 159 patients with cavity on chest radiograph were enrolled. Among them, 58 (36.5%) received standard treatment and 101 (63.5%) received extended treatment. The extended treatment group had higher AFB smear positivity than the standard group (56.4% vs. 29.3%, respectively, p = 0.002). However, apart from a lower prevalence of chronic kidney disease, there were no significant differences in demographic characteristics or other comorbidities between the two groups (Table 1).

**Table 1:** Baseline characteristics of pulmonary tuberculosis patients with cavity on chest radiograph.

Characteristics	Total, n = 159	Standard, n = 58	Extended, n = 101	P value
Age (years)	54.75 ± 14.29	56.40 ± 14.07	53.80 ± 14.40	0.269
Males, n (%)	127 (79.9%)	47 (81.0%)	80 (79.2%)	0.943
BMI < 18.5 kg/m^2^, n/N (%)	26/136 (19.1%)	5/44 (11.4%)	21/92 (22.8%)	0.174
Ever smoker, n (%)	113(71.1%)	42 (72.4%)	71 (70.3%)	0.919
Comorbid conditions				
Hypertension, n (%)	33 (20.8%)	14 (24.1%)	19 (18.8%)	0.552
Diabetes mellitus, n (%)	49 (30.8%)	16 (27.6%)	33 (32.7%)	0.624
Cardiovascular disease, n (%)	2 (1.3%)	1 (1.7%)	1 (1.0%)	>0.999
Chronic pulmonary disease, n (%)	7 (4.4%)	4 (6.9%)	3 (3.0%)	0.447
Chronic liver disease, n (%)	5 (3.1%)	1 (1.7%)	4 (4.0%)	0.760
Chronic kidney disease, n (%)	3 (1.9%)	3 (5.2%)	0 (0.0%)	0.089
Cerebrovascular disease, n (%)	2 (1.3%)	1 (1.7%)	1 (1.0%)	>0.999
Malignancy, n (%)	3 (1.9%)	1 (1.7%)	2 (2.0%)	>0.999
Previous history of TB treatment, n (%)	28 (17.6%)	8 (13.8%)	20 (19.8%)	0.459
Extrapulmonary TB, n (%)	11 (6.9%)	4 (6.9%)	7 (6.9%)	>0.999
Multi-lobe infiltration, n (%)	71 (44.7%)	25 (43.1%)	46 (45.5%)	0.894
AFB smear positivity, n (%)	74 (46.5%)	17 (29.3%)	57 (56.4%)	0.002

Abbreviations: AFB: acid-fast bacilli; BMI: body mass index; TB: tuberculosis.

Baseline laboratory abnormalities were additionally compared between the two groups and are presented in S1 Table. Baseline leukopenia was more frequent in the standard-treatment group than in the extended-treatment group, whereas the frequencies of anemia, thrombocytopenia, abnormal liver function, and abnormal renal function were not significantly different between groups.

Recurrence did not differ between the two groups (3.4% in the standard group vs. 2.0% in the extended group, p = 0.966), nor did the rate of 2-month culture positivity (6.3% vs. 4.4%, respectively, p > 0.999). In addition, no significant difference was observed in the time to culture conversion between the groups (8.00 ± 3.91 weeks vs. 7.45 ± 3.73 weeks, respectively, p = 0.454) (Table 2).

**Table 2:** Comparison of treatment outcomes between standard and extended treatment in pulmonary tuberculosis with cavity on chest radiograph.

Variables	Total, N = 159	Standard, n = 58	Extended, n = 101	p value
Treatment duration (days)	251.75 ± 68.47	181.98 ± 13.31	291.81 ± 53.50	<0.001
Duration of follow up after treatment completion (days)	404.10 ± 191.34	414.48 ± 223.51	398.14 ± 171.09	0.631
2-month culture positivity, n/N (%)	4/77 (5.2%)	2/32 (6.3%)	2/45 (4.4%)	>0.999
Time to culture conversion (weeks)	7.66 ± 3.79	8.00 ± 3.91	7.45 ± 3.73	0.454
Treatment interruption of > 7 days for any reason, n (%)	13(8.2%)	4 (6.9%)	9 (8.9%)	0.884
Recurrence, n (%)	4 (2.5%)	2 (3.4%)	2 (2.0%)	0.966

Among the four patients with bacteriologically confirmed recurrence, two were in the standard-treatment group and two were in the extended-treatment group. In the standard-treatment group, recurrence was detected 197 days (6.5 months) and 414 days (13.6 months) after treatment completion. In the extended-treatment group, recurrence was detected 295 days (9.7 months) and 378 days (12.4 months) after treatment completion. Because molecular typing was not available, relapse and reinfection could not be definitively distinguished. Based on timing alone, the earlier recurrences were clinically more compatible with relapse, whereas the later recurrences could represent either late relapse or reinfection (S2 Table).

Medication interruption due to adverse drug events tended to be more frequent in the extended group. However, there was no significant difference in the overall incidence of adverse events (Table 3).

**Table 3:** Adverse drug reactions according to treatment duration in pulmonary tuberculosis patients with cavity on chest radiograph.

Variables	Total, N = 159	Standard, n = 58	Extended, n = 101	p value
Gastrointestinal adverse reaction^a^, n (%)	63 (39.6%)	21(36.2%)	42 (41.6%)	0.618
Hepatotoxicity^b^, n (%)	47(29.6%)	19(32.8%)	28 (27.7%)	0.625
Cutaneous adverse drug reaction^c^, n (%)	52(32.7%)	19(32.8%)	33(32.7%)	>0.999
Leukopenia^d^, n (%)	14 (8.8%)	3(5.2%)	11(10.9%)	0.350
Generalized weakness^e^, n (%)	34(21.4%)	15(25.9%)	19(18.8%)	0.399
Ototoxicity^f^, n (%)	11(6.9%)	4 (6.9%)	7 (6.9%)	>0.999
Nephrotoxicity^g^, n (%)	5 (3.1%)	3 (5.2%)	2 (2.0%)	0.523
Infection^h^, n (%)	10 (6.3%)	4 (6.9%)	6 (5.9%)	>0.999
Treatment interruption due to adverse drug reactions, n (%)	9 (5.7%)	1 (1.7%)	8 (7.9%)	0.204

^a^ Defined as the occurrence of nausea, vomiting, diarrhea, abdominal discomfort, or anorexia

^b^ Defined as elevation of AST, ALT, or total bilirubin above the institutional upper limit of normal

^c^ Defined as newly developed rash, pruritus, urticaria, or drug eruption

^d^ White blood cell count < 4,000 cells/µL

^e^ Defined as newly documented generalized weakness during anti-tuberculosis treatment that was assessed by the treating physician and recorded in the medical records.

^f^ Defined as newly documented hearing impairment, tinnitus, vertigo, or vestibular symptoms considered related to anti-tuberculosis treatment

^g^ Defined as serum creatinine elevation above the institutional upper limit of normal or ≥1.5 times baseline

^h^ Defined as a newly diagnosed infection during anti-tuberculosis treatment, supported by clinical, laboratory, radiologic, or microbiologic evidence and/or antimicrobial treatment.

In univariable analyses using logistic regression, no baseline demographic, clinical, radiologic, or microbiologic variables were significantly associated with recurrence. Diabetes mellitus, chronic pulmonary disease, and thrombocytopenia showed nonsignificant trends toward higher recurrence, but confidence intervals were wide due to the small number of events (S3 Table).

However, in Firth’s penalized logistic regression analysis for recurrence using a 200-day fixed-time approach, the exploratory adjusted analysis yielded an adjusted odds ratio of 0.07 for extended treatment, but this association did not reach statistical significance (95% CI, 0.01–1.06; p = 0.056). In contrast, low body mass index (BMI) (<18.5 kg/m²) was associated with recurrence in the multivariable Firth's penalized logistic regression model (OR, 81.92; 95% CI, 4.95–21,694.24; p < 0.001), although this estimate was highly imprecise due to sparse data (Table 4).

**Table 4:** Firth's penalized logistic regression analysis of factors associated with recurrence in pulmonary tuberculosis patients with cavity on chest radiograph using a 200-day fixed-time approach.

Variables	Univariate			Multivariate ^b^		
	OR	95% CI	p value	OR	95% CI	p value
Age ≥ 65 years	1.26	0.12-7.96	0.816	0.33	<0.01 −5.46	0.483
Males	2.36	0.24-316.87	0.522	5.12	0.24 −1797.41	0.346
Ever smoker	0.96	0.15-10.11	0.968			
BMI < 18.5 kg/m^2^	32.91	3.05-4482.79	0.002	81.92	4.95 −21694.24	<0.001
Hypertension	0.41	<0.01-3.96	0.502			
Diabetes mellitus	5.49	0.88-57.83	0.069			
Cardiovascular disease	6.82	0.05-102.73	0.327			
Chronic pulmonary disease	9.86	0.87-71.76	0.062			
Chronic liver disease	2.36	0.02-28.52	0.528			
Chronic kidney disease	4.84	0.03-63.02	0.400			
Cerebrovascular disease	6.82	0.05-102.73	0.327			
Malignancy	4.84	0.03-63.02	0.399			
Previous history of TB treatment	2.00	0.19-12.76	0.507			
Extrapulmonary TB	1.40	0.01-14.43	0.833			
Multi-lobe infiltration	1.24	0.19-8.25	0.809			
AFB smear positivity	1.15	0.17-7.63	0.876			
2 months positivity ^a^	—	—	>0.999			
Extended treatment	0.57	0.09-3.77	0.536	0.07	0.01 −1.06	0.056

Abbreviations: OR: odds ratio; CI: confidence interval; BMI: body mass index; TB: tuberculosis; AFB: acid-fast bacilli

^a^ Estimates were unstable (OR extremely large with infinite/undefined CI) due to sparse data and quasi-complete separation, and thus not reported.

^b^ The multivariable model included extended treatment, age, sex, and BMI. Because BMI data were missing in 23 patients, this model was conducted as a complete-case analysis without imputation and included 136 patients with 3 recurrence events.

Sensitivity analyses were performed to assess the robustness of the association between extended treatment and recurrence. When alternative thresholds were applied to define extended treatment duration using a 200-day fixed-time approach (≥180, ≥ 200, and ≥220 days), the results were consistent, with extended treatment showing a numerically lower point estimate for recurrence across all definitions, although none reached statistical significance (S4 Table). In addition, analyses using alternative model specifications with varying degrees of covariate adjustment showed a similar direction of association (S5 Table). Because diabetes mellitus and chronic pulmonary disease showed possible associations with recurrence in the univariable analysis, additional sensitivity analyses were performed by including these variables in the multivariable model. The direction of the association between extended treatment and recurrence remained consistent across these models, although estimates remained imprecise because of the small number of recurrence events (S6 Table).

## Discussion

In this multicenter Korean cohort of DS pulmonary TB patients with cavity on chest radiograph who achieved treatment success with the HRZE regimen, a 200-day fixed-time approach combined with Firth’s penalized logistic regression was applied to address sparse events, and extended treatment beyond 200 days was not statistically significantly associated with a reduced risk of recurrence. Although the point estimate favored extended treatment, the analysis was based on only a small number of recurrence events and should therefore be interpreted cautiously. This finding is important for several reasons.

First, to the best of our knowledge, there is a lack of studies that have directly compared the risk of recurrence according to treatment duration among DS pulmonary TB patients with cavity on chest radiograph, including within existing guideline frameworks. Although current guidelines recommend treatment extension in selected high-risk patients, the evidence directly evaluating the incremental benefit of extended treatment duration on recurrence prevention remains limited. In this context, our study provides meaningful real-world evidence by explicitly examining the association between treatment duration and recurrence in a contemporary multicenter cohort.

Second, by directly comparing patients who received standard treatment with those who underwent extended treatment, our study provides an opportunity to explore objective evidence regarding the potential benefit of treatment extension. In routine clinical practice, decisions to extend therapy are largely influenced by perceived baseline severity and clinician judgment regarding response [7,8,15,16]. As observed in our study, baseline AFB smear positivity was substantially higher in the extended-treatment group than in the standard-treatment group (56.4% vs. 29.3%; p = 0.002), suggesting that treatment extension was selectively applied based on perceived bacillary burden rather than uniformly determined by guideline-based criteria. Treatment extension based primarily on clinical judgment, in the absence of objective supporting evidence, may inadvertently result in unnecessary extension of treatment, with potential implications for drug-related toxicity, compromised treatment adherence, and increased healthcare resource utilization. Especially, anti-TB therapy is associated with clinically relevant adverse drug reactions, including hepatotoxicity, and longer exposure increases time-at-risk and monitoring burden [17–20]. In our cohort, treatment interruption due to adverse reactions was numerically higher in the extended-treatment group (1.7% vs. 7.9%; p = 0.204). Although imprecise, this pattern is compatible with the expectation that cumulative exposure increases the probability of clinically significant intolerance and associated interruptions [21].

In this context, our findings underscore the need to identify and select high-risk individuals when considering treatment extension. These results support a more selective, evidence-based approach—prioritizing the identification of high-risk patients rather than relying solely on clinician-driven decision-making, thereby potentially avoiding unnecessary extension in lower-risk patients.

Lastly, in addition to extended treatment, we observed that low BMI was significantly associated with tuberculosis recurrence. This finding is biologically and clinically plausible, as low BMI reflects undernutrition and impaired host immunity, both of which are well-established risk factors for poor treatment response and disease recurrence in tuberculosis. Previous studies have consistently shown that underweight patients have delayed sputum conversion, reduced bactericidal activity, and higher rates of post-treatment recurrence, likely due to compromised cell-mediated immunity and altered pharmacokinetics of anti-tuberculosis drugs [22,23]. Our results are therefore concordant with existing evidence and suggest that nutritional status may be clinically relevant to long-term treatment outcomes. However, because this estimate was based on only four recurrence events and had a very wide confidence interval, the magnitude of the association should be interpreted with caution.

Our study has several limitations. First, the retrospective design, as opposed to a randomized prospective study, introduces inherent limitations such as selection bias and unmeasured confounding, despite our efforts to adjust for known covariates. In addition, our analytic cohort was restricted to patients with DS pulmonary tuberculosis who achieved treatment success with the standard HRZE regimen. Therefore, our findings should be interpreted as comparing recurrence after treatment completion among patients who successfully completed treatment, rather than as estimating the overall efficacy or net clinical benefit of extended treatment. This selection may have introduced survivorship bias and may have underestimated potential harms or competing risks associated with prolonged treatment. Further prospective studies including all eligible patients from treatment initiation are needed to better evaluate the net clinical benefit of extended treatment.

Second, the low number of recurrence events represents a major limitation of this study. Bacteriologically confirmed recurrence was uncommon, with only four events observed (4/159, 2.5%). Programmatic factors may have contributed to this low recurrence frequency; in Korea, public–private mix–supported tuberculosis care has been associated with improved adherence support and systematic follow-up, which may reduce both on-treatment complications and post-treatment recurrence [24]. Consequently, this study was underpowered to draw definitive conclusions regarding the effect of extended treatment on recurrence, and the effect estimates are expected to be unstable, as reflected by the wide confidence interval. To mitigate this issue, we applied Firth’s penalized logistic regression, which is specifically designed to reduce small-sample bias and provide more reliable estimates in the presence of rare events. Nevertheless, even with this approach, the extremely small number of events limits the robustness and generalizability of the findings, and the results should be interpreted as exploratory rather than definitive. For the same reason, time-dependent Cox modeling or continuous treatment-duration modeling could not be reliably applied in the present study, as these approaches would have produced highly unstable estimates. This limitation also necessitates careful contextual interpretation in comparison with prior evidence and existing guideline frameworks. Current recommendations emphasize treatment extension particularly for patients with both baseline cavity and persistent culture positivity at two months, a combined phenotype consistently associated with a higher risk of recurrence in previous studies [1,3–10]. In contrast, our study evaluated a broader population defined by cavity on chest radiograph, rather than the narrower subgroup characterized by both cavity and delayed culture conversion. Moreover, two-month culture positivity was rare in our cohort among patients with available results (4/77, 5.2%), which limited our ability to adequately assess outcomes in the guideline-defined highest-risk subgroup.

Because the observed effect estimate was based on only four recurrence events and was therefore unstable, we did not base the future sample size estimation on the observed odds ratio. For planning purposes, assuming a clinically meaningful reduction in recurrence from 5% to 1%, a two-sided α of 0.05, 80% power, and 1:1 allocation, a future study would require approximately 285 patients per group, or 570 patients in total. These findings underscore the need for a substantially larger study or a cohort enriched for patients at higher recurrence risk, such as those with both baseline cavitation and persistent culture positivity at two months.

Third, there were missing data for variables associated with the outcome. Baseline BMI data were missing for 23 of 159 patients (14.5%), primarily because height and/or weight information was not available in the cohort records. Missing BMI data were more frequent in the standard-treatment group than in the extended-treatment group (24.1% vs. 8.9%; p = 0.017). Given the strong association between low BMI and recurrence, this differential missingness may have introduced selection bias and affected the precision and stability of the model estimates. Therefore, the association between low BMI and recurrence should be interpreted with caution.

Similarly, although sputum smear and culture examinations were intended to be performed according to the cohort protocol, two-month culture data were missing in 26 of 58 patients (44.8%) in the standard-treatment group and 56 of 101 patients (55.4%) in the extended-treatment group, with no statistically significant between-group difference (p = 0.249). Sensitivity analyses treating missing two-month culture results as either negative or positive showed no statistically significant between-group difference in two-month culture positivity, and univariable logistic analyses showed no significant association between two-month culture positivity and recurrence under these assumptions (S7 Table). However, these analyses cannot fully overcome the limitation caused by the high proportion of missing two-month culture data; therefore, the prognostic importance of two-month culture positivity and the treatment effect in the guideline-defined subgroup of patients with both baseline cavitation and persistent culture positivity at 2 months could not be reliably assessed.

Fourth, treatment extension in our cohort was not governed by predefined protocol-driven criteria, but was determined by the clinical judgment of the treating physicians at each participating center. As a result, patients selected for extended treatment may have had greater baseline disease burden or slower treatment response. Consistent with this possibility, the extended-treatment group had a significantly higher proportion of baseline AFB smear positivity than the standard-treatment group. In addition, the specific reasons for treatment extension, such as perceived disease severity, delayed clinical response, adherence concerns, or institutional practice patterns, were not systematically captured in the case report forms or available medical records and therefore could not be reliably reconstructed retrospectively. Thus, the similar recurrence rates observed between groups should not be interpreted as evidence of equivalence or absence of benefit, because they may reflect either a true protective effect of extended treatment offsetting higher baseline risk, or residual confounding by indication. Although methods such as propensity score matching or inverse probability of treatment weighting may be useful in larger observational cohorts, they were not applied in the present study because the small sample size and only four recurrence events would likely result in unstable propensity models or extreme weights.

Fifth, although we adjusted for measured covariates, residual confounding is likely to have remained. In particular, detailed radiologic severity related to cavitary disease burden could not be fully captured in our analysis. Current guideline recommendations are based primarily on the presence or absence of cavitation on chest radiography, rather than detailed characterization of cavitary burden [8]. Consistent with this framework, our cohort captured the presence of cavitation and information on multilobar infiltration as an indicator of overall disease extent. However, previous studies have reported that cavitation-specific features, including the number and size of cavities and multilobar involvement, may serve as indicators of tuberculosis disease burden and may be associated with treatment response and recurrence risk.[6–8,10,25,26]. In our cohort, detailed cavitation-specific variables, such as cavity size, number of cavities, and the extent of cavitary involvement, were not systematically recorded and therefore could not be incorporated into the analysis. The absence of these detailed radiologic data limits our ability to fully adjust for baseline disease severity and may have contributed to residual confounding. Other clinically relevant factors that may have contributed to residual confounding include symptom burden, treatment adherence quality, drug exposure and pharmacokinetics, clinicians’ subjective assessment of treatment response, and additional features plausibly associated with recurrence risk, such as extensive disease, substantial treatment interruptions, immunosuppression, and poorly controlled diabetes [5,9,27].

Sixth, our study design is subject to limitations related to outcome and exposure definitions. The definition of the outcome as bacteriologically confirmed recurrence during the documented post-treatment follow-up period improves specificity but may lead to under-ascertainment if microbiologic testing is not performed at recurrence, if recurrence is diagnosed clinically without culture confirmation, or if recurrence occurred outside the observed follow-up period. Post-treatment recurrence may reflect either relapse or reinfection, and the relative contribution of reinfection may increase with longer intervals after treatment completion [28]. Because molecular typing was also not available, we were unable to distinguish relapse from reinfection. As treatment extension would be expected to preferentially reduce relapse rather than reinfection, the coexistence of these mechanisms may have attenuated the observed association between extended treatment and recurrence risk [29].

Lastly, the use of 200 days as the threshold for defining extended treatment was a pragmatic choice. Although 6 months of daily therapy corresponds to approximately 180 days, real-world practice involves scheduling variability, brief treatment interruptions, and administrative delays. The 200-day cutoff was intended to distinguish minor deviations from more intentional treatment extension [15]; nevertheless, some degree of exposure misclassification is possible. Future studies could incorporate measures of cumulative drug exposure or adherence (e.g., proportion of doses taken), model treatment duration as a continuous variable, or apply flexible methods to explore potential non-linear associations.

## Conclusion

In this cohort of DS pulmonary TB patients with cavity on chest radiograph who successfully completed treatment, the small number of recurrence events limited our ability to definitively determine whether extending therapy beyond 200 days reduces bacteriologically confirmed recurrence. Although the adjusted estimate favored extended treatment, it was imprecise and should be interpreted as exploratory. Larger prospective studies are needed to clarify which patients with cavitary disease may benefit from individualized treatment extension.

## Supporting information

S1 Table Comparison of baseline laboratory abnormalities between the standard- and extended-treatment groups. (DOCX)

S2 Table Clinical description of bacteriologically confirmed recurrence cases. (DOCX)

S3 Table Univariable logistic regression analysis of baseline variables with recurrence counts. (DOCX)

S4 Table Sensitivity analysis using alternative definitions of extended treatment in a 200-day fixed-time Firth's penalized logistic regression model. (DOCX)

S5 Table Sensitivity analyses using alternative model specifications. (DOCX)

S6 Table Sensitivity analyses of the association between extended treatment and recurrence using alternative covariate adjustments. (DOCX)

S7 Table Sensitivity analyses for missing two-month culture results. (DOCX)
