# Supplementary material for: Extended treatment duration and recurrence in successfully treated drug-susceptible pulmonary tuberculosis with cavity on chest radiograph
Source: PLoS One. 2026 Sep 10;21(9):e0357772. doi: 10.1371/journal.pone.0357772 (PMC13561312; doi:10.1371/journal.pone.0357772)
Supplement: Supplementary file 1 [file pone.0357772.s001.docx]

**Supporting Table S1. Comparison of baseline laboratory abnormalities between the standard- and extended-treatment groups**

| **Variables** | **Total, N = 159** | **Standard, n = 58** | **Extended, n = 101** | **p value** |
| --- | --- | --- | --- | --- |
| Leukopeniaᵃ, n/N (%) | 23/159 (14.5) | 13/58 (22.4) | 10/101 (9.9) | 0.031 |
| Anemiaᵇ, n/N (%) | 68/141 (48.2) | 24/47 (51.1) | 44/94 (46.8) | 0.634 |
| Thrombocytopeniaᶜ, n/N (%) | 6/141 (4.3) | 4/47 (8.5) | 2/94 (2.1) | 0.095 |
| Abnormal liver functionᵈ, n/N (%) | 23/139 (16.5) | 8/47 (17.0) | 15/92 (16.3) | 0.914 |
| Abnormal renal functionᵉ, n/N (%) | 15/141 (10.6) | 8/47 (17.0) | 7/94 (7.4) | 0.082 |

^a^ White blood cell count <4,000 cells/µL

^b^ Haemoglobin level < 10.0 g/dL

^c^ Platelet count < 150,000/µL

^d^ AST > 40 U/L, ALT > 40 U/L, or total bilirubin > 1.3 mg/dL

^e^ BUN > 20 mg/dL and creatinine > 1.3 mg/dL.
