# Supplementary material for: Extended treatment duration and recurrence in successfully treated drug-susceptible pulmonary tuberculosis with cavity on chest radiograph
Source: PLoS One. 2026 Sep 10;21(9):e0357772. doi: 10.1371/journal.pone.0357772 (PMC13561312; doi:10.1371/journal.pone.0357772)
Supplement: Supplementary file 2 [file pone.0357772.s002.docx]

**Supporting Table S2. Clinical description of bacteriologically confirmed recurrence cases**

| **Case** | **Treatment group** | **Time from treatment completion to recurrence** | **Timing category** | **Suspected relapse vs. reinfection** |
| --- | --- | --- | --- | --- |
| **Case 1** | Standard treatment | 197 days (6.5 months) | Earlier post-treatment recurrence | More compatible with relapse based on timing |
| **Case 2** | Standard treatment | 414 days (13.6 months) | Later post-treatment recurrence | Late relapse or reinfection |
| **Case 3** | Extended treatment | 295 days (9.7 months) | Earlier post-treatment recurrence | More compatible with relapse based on timing |
| **Case 4** | Extended treatment | 378 days (12.4 months) | Later post-treatment recurrence | Late relapse or reinfection |

Note. All four cases were bacteriologically confirmed recurrences. Molecular typing was not available; therefore, relapse and reinfection could not be definitively distinguished. The suspected mechanism was interpreted cautiously based on timing after treatment completion only.

Abbreviation: TB, tuberculosis.
