# Supplementary material for: Extended treatment duration and recurrence in successfully treated drug-susceptible pulmonary tuberculosis with cavity on chest radiograph
Source: PLoS One. 2026 Sep 10;21(9):e0357772. doi: 10.1371/journal.pone.0357772 (PMC13561312; doi:10.1371/journal.pone.0357772)
Supplement: Supplementary file 3 [file pone.0357772.s003.docx]

**Supporting Table S3. Univariable logistic regression analysis of baseline variables with recurrence counts**

| Variable | level | Recurrence (n/N, %) | OR (95% CI) | p-value |
| --- | --- | --- | --- | --- |
| Age ≥ 65 years | No | 3/119 (2.5) | Reference |  |
|  | Yes | 1/40 (2.5) | 0.99 (0.05–8.00) | 0.994 |
| Males | No | 0/32 (0.0) | Reference |  |
|  | Yes | 4/127 (3.1) | NA (NA–NA) | 0.993 |
| Ever smoker | No | 1/46 (2.2) | Reference |  |
|  | Yes | 3/113 (2.7) | 1.23 (0.15–25.17) | 0.861 |
| BMI < 18.5 kg/m^2^ | No | 0/110 (0.0) | Reference |  |
|  | Yes | 3/26 (11.5) | NA (NA–NA) | 0.994 |
| Hypertension | No | 4/126 (3.2) | Reference |  |
|  | Yes | 0/33 (0.0) | NA (NA–NA) | 0.996 |
| Diabetes mellitus | No | 1/110 (0.9) | Reference |  |
|  | Yes | 3/49 (6.1) | 7.11 (0.88–145.78) | 0.093 |
| Cardiovascular disease | No | 4/157 (2.5) | Reference |  |
|  | Yes | 0/2 (0.0) | NA (NA–NA) | 0.996 |
| Chronic pulmonary disease | No | 3/152 (2.0) | Reference |  |
|  | Yes | 1/7 (14.3) | 8.28 (0.38–77.02) | 0.085 |
| Chronic liver disease | No | 4/154 (2.6) | Reference |  |
|  | Yes | 0/5 (0.0) | NA (NA–NA) | 0.996 |
| Chronic kidney disease | No | 4/156 (2.6) | Reference |  |
|  | Yes | 0/3 (0.0) | NA (NA–NA) | 0.995 |
| Cerebrovascular disease | No | 4/157 (2.5) | Reference |  |
|  | Yes | 0/2 (0.0) | NA (NA–NA) | 0.996 |
| Malignancy | No | 4/156 (2.6) | Reference |  |
|  | Yes | 0/3 (0.0) | NA (NA–NA) | 0.995 |
| Previous history of TB treatment | No | 3/131 (2.3) | Reference |  |
|  | Yes | 1/28 (3.6) | 1.58 (0.08–12.88) | 0.697 |
| Extrapulmonary TB | No | 4/148 (2.7) | Reference |  |
|  | Yes | 0/11 (0.0) | NA (NA–NA) | 0.994 |
| Multi-lobe infiltration | No | 2/88 (2.3) | Reference |  |
|  | Yes | 2/71 (2.8) | 1.25 (0.15–10.60) | 0.828 |
| AFB smear positivity | No | 2/85 (2.4) | Reference |  |
|  | Yes | 2/74 (2.7) | 1.15 (0.14–9.81) | 0.888 |
| Leukopeniaᵃ | No | 4/136 (2.9) | Reference |  |
|  | Yes | 0/23 (0.0) | NA (NA–NA) | 0.994 |
| Anemiaᵇ | No | 0/73 (0.0) | Reference |  |
|  | Yes | 4/68 (5.9) | NA (NA–NA) | 0.993 |
| Thrombocytopeniaᶜ | No | 3/135 (2.2) | Reference |  |
|  | Yes | 1/6 (16.7) | 8.80 (0.40–85.00) | 0.080 |
| LFT elevationᵈ | No | 3/116 (2.6) | Reference |  |
|  | Yes | 1/23 (4.3) | 1.71 (0.08–14.08) | 0.648 |
| RFT elevationᵉ | No | 3/126 (2.4) | Reference |  |
|  | Yes | 1/15 (6.7) | 2.93 (0.14–24.70) | 0.366 |

Abbreviations: OR: odds ratio; CI: confidence interval; BMI: body mass index; TB: tuberculosis; AFB: acid-fast bacilli; AE: adverse events; NA: not available.

^a^ White blood cell count <4,000 cells/µL.

^b^ Haemoglobin level < 10.0 g/dL.

^c^ Platelet count < 150,000/µL.

^d^ AST > 40 U/L, ALT > 40 U/L, or total bilirubin > 1.3 mg/dL.

^e^ BUN > 20 mg/dL and creatinine > 1.3 mg/dL.
