# Supplementary material for: Extended treatment duration and recurrence in successfully treated drug-susceptible pulmonary tuberculosis with cavity on chest radiograph
Source: PLoS One. 2026 Sep 10;21(9):e0357772. doi: 10.1371/journal.pone.0357772 (PMC13561312; doi:10.1371/journal.pone.0357772)
Supplement: Supplementary file 4 [file pone.0357772.s004.docx]

**Supporting Table S4. Sensitivity analysis using alternative definitions of extended treatment in a 200-day fixed-time Firth penalized logistic regression model**

| Settings | n | events | OR (95% CI) | p |
| --- | --- | --- | --- | --- |
| Extended treatment ≥180 days | 159 | 4 | 0.74 (0.02–144.25) | 0.879 |
| Extended treatment ≥200 days | 159 | 4 | 0.07 (<0.01–1.06) | 0.056 |
| Extended treatment ≥220 days | 159 | 4 | 0.06 (<0.01–1.04) | 0.056 |

Abbreviations: OR: odds ratio; CI: confidence interval.
