# Supplementary material for: Extended treatment duration and recurrence in successfully treated drug-susceptible pulmonary tuberculosis with cavity on chest radiograph
Source: PLoS One. 2026 Sep 10;21(9):e0357772. doi: 10.1371/journal.pone.0357772 (PMC13561312; doi:10.1371/journal.pone.0357772)
Supplement: Supplementary file 5 [file pone.0357772.s005.docx]

**Supporting Table S5. Sensitivity analyses using alternative model specifications**

| **Models** | **n** | **events** | **OR (95% CI)** | **p** |
| --- | --- | --- | --- | --- |
| Main model | 159 | 4 | 0.07 (0.01–1.06) | 0.056 |
| Age-adjusted | 159 | 4 | 0.57 (0.09–3.80) | 0.539 |
| BMI-adjusted | 159 | 4 | 0.11 (0.01–1.03) | 0.053 |
| Unadjusted | 159 | 4 | 0.57 (0.09–3.77) | 0.536 |

Abbreviations: OR: odds ratio; CI: confidence interval; BMI: body mass index.
