# Supplementary material for: Extended treatment duration and recurrence in successfully treated drug-susceptible pulmonary tuberculosis with cavity on chest radiograph
Source: PLoS One. 2026 Sep 10;21(9):e0357772. doi: 10.1371/journal.pone.0357772 (PMC13561312; doi:10.1371/journal.pone.0357772)
Supplement: Supplementary file 6 [file pone.0357772.s006.docx]

**Supporting Table S6. Sensitivity analyses of the association between extended treatment and recurrence using alternative covariate adjustments**

| **Model** | **OR for extended treatment (95% CI)** | **p-value** |
| --- | --- | --- |
| Primary model | 0.07 (0.01–1.06) | 0.056 |
| Primary model + diabetes mellitus | 0.11 (<0.01–1.44) | 0.094 |
| Primary model + chronic pulmonary disease | 0.09 (<0.01–1.96) | 0.125 |
| Primary model + diabetes mellitus + chronic pulmonary disease | 0.12 (<0.01–2.20) | 0.149 |

Abbreviations: OR: odds ratio; CI: confidence interval.
