# Supplementary material for: Extended treatment duration and recurrence in successfully treated drug-susceptible pulmonary tuberculosis with cavity on chest radiograph
Source: PLoS One. 2026 Sep 10;21(9):e0357772. doi: 10.1371/journal.pone.0357772 (PMC13561312; doi:10.1371/journal.pone.0357772)
Supplement: Supplementary file 7 [file pone.0357772.s007.docx]

**Supporting Table S7. Sensitivity analyses for missing two-month culture results**

| **Analysis** | **2-month culture positivity by treatment group** | **p value** | **Crude OR for recurrence** | **p value** |
| --- | --- | --- | --- | --- |
| Complete-case analysis | Standard: 2/32 (6.3%); Extended: 2/45 (4.4%) | >0.999 | Not estimable | >0.999 |
| Missing results treated as negative | Standard: 2/58 (3.4%); Extended: 2/101 (2.0%) | 0.623 | Not estimable | >0.999 |
| Missing results treated as positive | Standard: 28/58 (48.3%); Extended: 58/101 (57.4%) | 0.265 | 0.85 | >0.999 |

Crude odds ratios were not estimable when recurrence events were absent in one or more comparison strata.

Abbreviations: OR: odds ratio.
