# Supplementary material for: Extended treatment duration and recurrence in successfully treated drug-susceptible pulmonary tuberculosis with cavity on chest radiograph
Source: PLoS One. 2026 Sep 10;21(9):e0357772. doi: 10.1371/journal.pone.0357772 (PMC13561312; doi:10.1371/journal.pone.0357772)
Supplement: Supplementary file 8 [file pone.0357772.s009.docx]

Response to the editor and reviewers

Ref: Submission ID PONE-D-26-05397

Title: Efficacy of Extended Treatment in Drug-Susceptible Pulmonary Tuberculosis with Cavity on Chest Radiograph

We sincerely thank the Editor and the Reviewer for their thoughtful and constructive feedback on our manuscript. We have carefully considered and addressed every comment in the revised version of the paper. Below, we present a detailed point-by-point response, with each reviewer’s comment followed by our corresponding reply. All changes have been incorporated into the manuscript, and the tracked-changes document highlights all new or substantially revised sections.

Reviewer #1: Major Comments

**1. Underpowered study and overstated conclusions**

**The primary analysis yielded an aOR of 0.07 (95% CI 0.01–1.06, p = 0.06), based on only four recurrence events, and did not reach statistical significance. Describing this as "a protective trend" risks overstating the evidence. The conclusion should more explicitly acknowledge that this study was underpowered to draw definitive conclusions. A post-hoc power calculation or sample size estimation for a future adequately powered study should be provided.**

We thank you for this important comment. We agree that the primary analysis, which was based on only four recurrence events, does not provide sufficient statistical evidence to support a definitive protective effect.

Accordingly, we revised the wording throughout the manuscript to avoid overstating the findings. First, in the Abstract and Results section, we removed the term “trend” from the description of the Firth penalized logistic regression analysis and replaced it with more objective wording (lines 97 and 289–292). Similarly, in the sensitivity analysis, we removed the phrase “protective trend” and revised the text using more objective language (lines 306-307).

In addition, at the beginning of the Discussion, we deleted the sentence containing the phrase “protective trend” to further avoid overstating the results (lines 318–324).

In addition, we have removed the statements in the Results section regarding the benefits of extended treatment to avoid the overstatement you pointed out. Instead, we have emphasized the potential risks of unnecessary treatment extension and its associated adverse effects, and revised the text to highlight the need for careful selection of patients who would require treatment extension in future practice. (Lines 351-355 in result section)

In the Limitations section, we also strengthened the statement that the small sample size limits the generalizability of the findings We replaced wording suggesting cautious interpretation with a clearer statement that the results should be interpreted as exploratory rather than definitive, thereby emphasizing the limited generalizability of the findings (Second paragraph in limitation section).

Furthermore, we added a description of the design considerations for a future study, including a specific sample size estimation, at the end of the second paragraph of the Limitations section.

Finally, consistent with Reviewer 2’s comment on the tone of the conclusion, we revised the concluding statement of the Discussion to avoid overstating our findings. We removed the phrase “suggest a potential benefit in selected patients” and instead emphasized that the study was underpowered, the adjusted estimate was imprecise, and the findings should be interpreted as exploratory. (Conclusion paragraph of the Discussion section)

In addition, as part of our revision and statistical verification process, we carefully re-examined the Firth penalized logistic regression results presented in Table 4. During this review, we noted that the originally calculated univariable estimates for age and sex were not accurately reflected in the previous version of the table. We sincerely apologize for this oversight and have corrected these values in the revised table. Importantly, the multivariable primary model results, including the adjusted estimate for extended treatment, remained unchanged, and these corrections do not affect the overall interpretation of the study findings.

**2. Absence of predefined indications for treatment extension**

**No protocol-defined criteria for extending treatment are described, and the Discussion acknowledges that extension was largely driven by clinician judgment. If the clinical reasons for treatment extension can be retrieved retrospectively, they should be reported. If not, this should be explicitly acknowledged as a limitation.**

Thank you for your insightful comment. We agree that treatment extension in our cohort was not governed by predefined protocol-driven criteria but was determined by the clinical judgment of the treating physicians at each participating center. However, the reasons for treatment extension were not documented in the cohort records and therefore could not be reliably retrieved retrospectively.

Accordingly, we have explicitly acknowledged this issue in the fourth limitation paragraph of the revised Discussion. We clarified that the absence of predefined indications for treatment extension and the inability to retrieve the specific rationale for each extension decision may have introduced residual confounding by indication.

**3. Radiologic severity of cavitation not captured**

**Cavity size, number, and extent of pulmonary involvement are recognized prognostic factors yet were not incorporated into the analysis. The authors should explore whether these data were recorded and, if so, include them in the analysis. If unavailable, this should be discussed more prominently as a source of residual confounding.**

We thank you for this important comment. As shown in our results, information on multilobar infiltration on chest radiography was available as an indicator of overall disease extent. However, this cohort did not include detailed radiologic data specifically characterizing cavitary disease burden, such as cavity size, number of cavities, or the extent of cavitary involvement. Therefore, these cavitation-specific severity measures could not be reliably retrieved retrospectively or incorporated into the analysis.

Accordingly, we expanded the fifth limitation paragraph of the revised Discussion to address this issue more explicitly. We noted that recent studies have suggested that cavitation-specific features, including cavity size, number of cavities, and extent of cavitary involvement, may serve as indicators of tuberculosis disease burden and recurrence risk, and we additionally cited the relevant supporting references. We also clarified that the lack of detailed radiologic information beyond the binary presence of cavitation may have limited our ability to fully adjust for baseline disease severity.

**4. Missing BMI data**

**BMI was the strongest independent predictor of recurrence, yet data were available for only 136 of 159 patients (85.5%). The reasons for and distribution of missing BMI data across groups should be reported, and the potential impact on model estimates should be addressed.**

We thank you for this very insightful comment.

(i) Reasons for missing BMI data:
The missing BMI values were attributable to the absence of height and/or weight information in the cohort records, rather than intentional exclusion. Unfortunately, no additional documentation was available to determine the specific reasons for these missing measurements.

(ii) Distribution of missing data across groups and potential impact on model estimates:
BMI was missing in 14 of 58 patients (24.1%) in the standard-treatment group and in 9 of 101 patients (8.9%) in the extended-treatment group, with a statistically significant difference between groups (p = 0.017). We agree that this imbalance is important, as complete-case analysis may have introduced selection bias. Given that BMI was identified as an important predictor of recurrence, differential missingness in BMI may also have affected the precision and stability of the model estimates.

Accordingly, we have added a new third paragraph to the Limitations section of the revised Discussion. In this paragraph, we describe the imbalance in missing BMI data between treatment groups and explicitly acknowledge that, because BMI was an important predictor of recurrence, this differential missingness may have introduced selection bias and should be considered when interpreting the findings.

**5. IRB approval for all participating institutions**

**Only a single IRB approval number is provided for an 18-center study. The authors should confirm that central IRB approval covered all sites or provide individual approval numbers for each institution.**

We thank the reviewer for this important comment. We confirm that the study was conducted in accordance with the Declaration of Helsinki and was approved by the Institutional Review Boards (IRBs) and Ethics Committees of all participating institutions. The study was coordinated and overseen by the representative institution, the Catholic University of Korea (IRB No. C19ONDI0458), which served as the coordinating center for the 17 other participating medical institutions. In accordance with the reviewer's suggestion, we have now provided the individual IRB approval numbers for each participating institution in the revised 'Ethics Statement' subsection of the Methods section, as follows:

The study was conducted in accordance with the Declaration of Helsinki and was approved by the Institutional Review Boards (IRBs) and Ethics Committees of all participating institutions. The study was coordinated and overseen by the representative institution, the Catholic University of Korea (IRB No. KC19ONDI0458), which represented and managed the study across the 17 other participating medical institutions. All participating institutions obtained IRB approval prior to study initiation, with the following approval numbers: SCHBC202012023001-HE001 (Soonchunhyang University Bucheon Hospital), UUH202103016-HE002 (Ulsan University Hospital), GCIRB2021-016 (Gachon University Gil Hospital), 2020GR0402 (Korea University Guro Hospital), VC19ONDI016 (St. Vincent's Hospital), KANGDONG2020-06-012-001 (Kangdong Sacred Heart Hospital), KYUH2020-12-013-002 (Konyang University Hospital), SCHCA202012076-HE005 (Soonchunhyang University Cheonan Hospital), DUIH202101023001-HE001 (Dongguk University Ilsan Hospital), 2020-07-007 (Chungnam National University Hospital), 2020-08-012-003 (Chungbuk National University Hospital), DC19ONDI0052 (Daejeon St. Mary's Hospital), HC19ONDI0068 (Bucheon St. Mary's Hospital), UC19ONDI0106 (Uijeongbu St. Mary's Hospital), PC19ONDI0149 (Eunpyeong St. Mary's Hospital), OC19ONDI0073 (Incheon St. Mary's Hospital), and ISPAIK2020-11-003-001 (Ilsan Paik Hospital). Written informed consent was obtained from all adult participants before their enrolment in the study.

**6. Sputum collection intervals**

**The timing and frequency of sputum collection are not described. Given that two-month culture positivity and culture conversion are key outcomes, and the number of patients with available data differed between groups (n = 32 vs. 45), the authors should confirm that sputum collection intervals were comparable between groups.**

We sincerely thank you for this insightful and important comment. We fully agree that the timing and frequency of sputum collection are important considerations when interpreting two-month culture positivity and culture conversion outcomes. According to the cohort protocol, sputum smear and culture examinations were intended to be performed as part of routine follow-up during anti-tuberculosis treatment, including assessment at approximately 2 months after treatment initiation. Therefore, the intended sputum collection schedule was comparable between the standard- and extended-treatment groups.

Nevertheless, two-month culture results were not available for all patients in this real-world multicenter cohort, despite the intended protocol-based follow-up schedule. This incomplete availability may have reflected several factors encountered in routine clinical practice, including inability to expectorate sputum after clinical improvement, variation in visit timing around the two-month assessment window, clinician discretion regarding follow-up testing, differences in institutional practice patterns, or incomplete capture of test results in the cohort records.

In response to this comment, and in addition to the issue of missing BMI data addressed in Comment 4, we further expanded the third limitation paragraph of the revised Discussion to address missing data for clinically important variables more broadly. Specifically, we acknowledged that the incomplete availability of two-month culture data may have limited our ability to fully evaluate the prognostic importance of two-month culture positivity and its association with recurrence.

**Minor Comments**

1. **The abstract should include a brief description of the source cohort.**

We thank you for this helpful comment. We have revised the Abstract to include a clearer description of the source cohort. Specifically, we clarified that this study was a retrospective analysis using data from a multicenter prospective cohort of patients with pulmonary tuberculosis enrolled at 18 Korean institutions, and that the present analysis focused on patients with drug-susceptible pulmonary tuberculosis and cavity on chest radiograph.

2. **Please explicitly state that only patients susceptible to all HRZE components were included, using the term “pan-susceptible” or equivalent, to avoid confusion with the current sequential exclusion criteria.**

Thank you for this helpful suggestion. To clarify the eligibility criteria and avoid confusion with the sequential exclusion criteria, we revised the Methods section to explicitly state that only patients with pan-susceptible tuberculosis were included. Specifically, we added that the study population was restricted to patients susceptible to all HRZE components (‘population’ subsection of Methods section)

**3. Specific patient numbers in the exclusion process should be moved from the Methods to the Results section or flow diagram legend.**

Thank you very much for this helpful and thoughtful suggestion. We agree that presenting the specific patient numbers for each exclusion step in the Results section or flow diagram is clearer and more appropriate than including them in the Methods section. Accordingly, we have revised the Methods section to describe the eligibility and exclusion criteria without specific patient numbers, and have moved the detailed exclusion counts to the Results section, with reference to Figure 1.

(‘Population’ Subsection of Method section)

(Result section: lines 226-234)

4. **"Gender" should be replaced with "sex" throughout the manuscript, consistent with ICMJE recommendations.**

Thank you very much for this important suggestion. We have revised the manuscript to replace “gender” with “sex” throughout, in accordance with ICMJE recommendations. (line 153)

5. **The rationale for the 200-day cutoff should be more explicitly justified in the Methods, with a clearer explanation of how the referenced study (ref 15) informed this threshold.**

Thank you very much for this valuable suggestion. We agree that the rationale for the 200-day cutoff should be more explicitly described to improve the clarity and reproducibility of our Methods. Accordingly, we have revised the Methods section to clarify that the cutoff was based on the standard 6-month regimen, corresponding to approximately 180 days, with an additional 2-week allowance. We also clarified that this allowance was informed by a previous study of prolonged TB treatment [15], which noted that treatment interruptions of ≥2 weeks during the intensive phase may require reintroduction of intensive-phase treatment and thereby prolong the total treatment duration. Thus, the 200-day cutoff was selected to distinguish meaningful treatment extension from minor variations around the standard 6-month course. (‘Group and study outcome’ subsection of Methods section: lines 182-189)

6. **The average duration of follow-up after treatment completion and its comparability between groups should be reported.**

Thank you very much for this helpful suggestion. We agree that reporting the follow-up duration after treatment completion is important for interpreting recurrence outcomes. The follow-up duration after treatment completion was comparable between the standard and extended treatment groups, with no statistically significant difference observed. These results have been added to Table 2.

7. **P-values in Supplementary Table 2 should be reported to at least three decimal places.**

Thank you very much for this insightful suggestion. We have revised the p-values to at least three decimal places, as requested. As the relevant table has been renumbered as Supplementary Table 4 in the revised manuscript, this change is now reflected in Supplementary Table 4. We also revised confidence interval values that had been rounded to 0.00 and reported them as <0.01 to avoid misinterpretation. In addition, we reviewed the entire manuscript and supplementary tables and standardized p-value reporting to three decimal places throughout.

**8. A brief clinical description of the four recurrence cases (timing, group assignment, suspected relapse vs. reinfection) would provide useful context for interpreting the primary outcome.**

Thank you very much for this important and very helpful comment. In carefully re-examining the four recurrence cases in response to your suggestion, we identified an inconsistency in our previous description of the primary outcome. Specifically, recurrence had been described as occurring “within 1 year after treatment completion”; however, two of the four recurrence cases were in fact detected more than 1 year after treatment completion.

We sincerely apologize for this oversight and are grateful for the opportunity to correct it. We would also like to respectfully clarify that, despite this wording error in the description of the primary outcome, all statistical analyses in the manuscript had been conducted based on recurrence events observed during the entire follow-up period, rather than being limited to recurrence within 1 year after treatment completion. Therefore, the correction pertains to the wording and definition of the outcome description, and does not affect the analytical approach or the reported results.

Accordingly, we have revised the outcome definition throughout the manuscript to refer to “bacteriologically confirmed recurrence during post-treatment follow-up,” rather than recurrence “within 1 year after treatment completion.” We have also added a brief clinical description of the four recurrence cases to the Results section. Among the four patients with bacteriologically confirmed recurrence, two were in the standard-treatment group and two were in the extended-treatment group. Recurrence was detected 197 and 414 days after treatment completion in the standard-treatment group, and 295 and 378 days after treatment completion in the extended-treatment group. Because molecular typing was not available, relapse and reinfection could not be definitively distinguished. Therefore, these cases were interpreted cautiously: the earlier recurrences were considered more compatible with relapse, whereas the later recurrences could represent either late relapse or reinfection.

In addition, we have added a new supplementary table summarizing the four recurrence cases as Supplementary Table S1. Accordingly, the numbering of the subsequent supplementary tables has been shifted sequentially.

**Reviewer #2: Dear Authors,**

**I appreciate the effort that went into this multicenter study evaluating the potential benefit of extended treatment in drug-susceptible pulmonary tuberculosis patients with cavitary disease.**

**The study addresses a clinically relevant and timely question, given the limited evidence base supporting current guideline recommendations for treatment extension.**

**The utilization of a well-established prospective cohort from 18 Korean institutions is a notable strength, and the manuscript is clearly written with an appropriate structure and length.**

**However, there are several methodological and interpretive aspects that require further clarification and discussion before the manuscript can be considered for publication. I have outlined my specific comments below.**

**1. Study Population and Potential Survivorship Bias**

**The study population is restricted to patients who achieved treatment success with the HRZE regimen, having excluded those who experienced treatment failure (n=4), death (n=53), loss to follow-up (n=8), and transfer to other clinics (n=41). This introduces a significant survivorship bias, as patients who received extended treatment but could not complete therapy due to adverse events or who died during treatment are systematically excluded from the analysis.**

**Consequently, the study does not evaluate the “efficacy of extended treatment” as the title implies, but rather compares recurrence rates among patients who successfully completed their respective treatment courses. This distinction is important and should be reflected in the title, abstract, and conclusions. Additionally, the Discussion section should include a dedicated paragraph addressing how this selection may have influenced the results.**

We sincerely thank you for this thoughtful and important comment. We agree that restricting the analytic cohort to patients who achieved treatment success may introduce survivorship bias and should be clearly acknowledged when interpreting our findings. As you correctly pointed out, our analysis does not estimate the overall efficacy or net clinical benefit of extended treatment among all patients who may have been considered for treatment extension. Rather, it compares recurrence after treatment completion among patients who successfully completed treatment. In this study, treatment completion was used as the starting point for assessing post-treatment recurrence, and a fixed-time approach was applied to reduce time-dependent bias in exposure classification. Nevertheless, this approach cannot fully address potential survivorship bias arising from the exclusion of patients who experienced treatment failure, died, were lost to follow-up, transferred to other clinics, or discontinued treatment before treatment completion.

In response to this comment, we revised the title to “Extended Treatment Duration and Recurrence in Successfully Treated Drug-Susceptible Pulmonary Tuberculosis with Cavity on Chest Radiograph” to more accurately reflect the analytic population. We have also revised the Discussion to explicitly acknowledge this limitation and to clarify the scope of interpretation of our findings. We agree that future studies should address this issue more comprehensively using prospective designs that include all eligible patients from treatment initiation and account for treatment failure, death, discontinuation, and other competing outcomes when evaluating the net clinical benefit of extended treatment. (First paragraph of Limitations, lines 370–378)

**2. Extremely Low Number of Recurrence Events and Model Stability**

**With only 4 recurrence events among 159 patients, the multivariable Firth’s penalized logistic regression model including 4 covariates (extended treatment, age, sex, and BMI) yields an events-per-variable ratio below 1.**

**While the choice of Firth’s method is appropriate to address separation, the fundamental limitation imposed by the extremely low event count cannot be fully overcome by any statistical technique.**

**This is clearly reflected in the confidence interval for low BMI (OR 81.92; 95% CI, 4.95–21,694.24), which spans over four orders of magnitude.**

**Characterizing this variable as an independent predictor may overstate the precision and reliability of the estimate.**

**While the direction of association is biologically plausible and consistent with prior literature, the magnitude should be interpreted with explicit caution.**

**It would be beneficial to add appropriate caveats both in the Results and Discussion sections**.

Thank you very much for this important and thoughtful comment. We fully agree with you that, although Firth’s penalized logistic regression is an appropriate method for reducing small-sample bias and addressing separation, it cannot overcome the fundamental limitation imposed by the very small number of recurrence events. We also agree that the very wide confidence interval for low BMI reflects substantial statistical imprecision, and that the magnitude of this association should therefore be interpreted with great caution.

In response to this comment, we have revised the Abstract, Results, and Discussion sections to avoid overstating the role of low BMI. Specifically, we replaced wording such as “independent predictor” and “independent determinant” with more cautious language, including “associated with recurrence” and “the clinical relevance of nutritional status.” We also added an explicit cautionary statement in the Discussion noting that, because the estimate was based on only four recurrence events and had a very wide confidence interval, the magnitude of the association should be interpreted with caution.

(Abstract: lines 99-100)

(Result section: lines 292-294)

(Discussion section: lines 365-367)

**3. Confounding by Indication**

**The extended treatment group had significantly higher baseline AFB smear positivity (56.4% vs. 29.3%, p<0.01), suggesting that clinicians selectively extended treatment for patients with perceived greater disease severity.**

**This introduces confounding by indication—a well-recognized limitation in observational treatment-duration studies.**

**If the extended treatment group had a higher baseline disease burden yet showed similar recurrence rates to the standard group, this observation could reflect either a genuine protective effect of extended treatment or residual confounding. The current analysis does not adequately address this issue. The authors should consider discussing the potential utility of methods such as propensity score matching or inverse probability of treatment weighting, and at minimum provide a thorough discussion of how confounding by indication may have affected the interpretation of results.**

Thank you very much for this important and insightful comment. We fully agree with you that confounding by indication is an important limitation of this observational treatment-duration study, particularly because treatment extension was determined by the clinical judgment of the treating physicians rather than by predefined protocol-driven criteria.

In response to this comment, we have revised the fourth limitation paragraph of the Discussion to address this issue more explicitly. Specifically, we now acknowledge that patients selected for extended treatment may have had greater baseline disease burden or slower treatment response, as suggested by the significantly higher proportion of baseline AFB smear positivity in the extended-treatment group. We also clarify that the similar recurrence rates observed between groups should not be interpreted as evidence of equivalence or absence of benefit, because this finding may reflect either a true protective effect of extended treatment offsetting higher baseline risk or residual confounding by indication.

We also carefully considered the use of propensity score matching or inverse probability of treatment weighting. However, given the small sample size and only four recurrence events, we did not apply these methods, as they would likely have resulted in unstable propensity models or extreme weights and therefore would not have reliably improved causal inference in the present study.

**4. Variable Selection in the Multivariable Model**

**The rationale for variable selection in the multivariable model is not fully transparent.**

**Age and sex were included a priori, and BMI was included based on clinical relevance and consistency with prior evidence. However, in the univariable analysis, diabetes mellitus (OR 5.49, p=0.07) and chronic pulmonary disease (OR 9.86, p=0.06) demonstrated associations with recurrence that were at least as strong as, or stronger than, several included variables, yet they were not selected for the final model.**

**Given the severe constraint imposed by the small number of events, it is understandable that not all variables could be included.**

**Nevertheless, the authors should provide explicit criteria for variable selection and consider presenting alternative model specifications with different covariate combinations in sensitivity analyses to demonstrate the robustness of the findings.**

Thank you very much for this important and constructive comment. We agree that the rationale for covariate selection should be clearly stated, particularly given the very small number of recurrence events and the resulting risk of model instability.

In response, we have revised the ‘Statistical analysis’ subsection of Methods section to provide explicit criteria for variable selection. We now clarify that, given the very small number of recurrence events, we sought to minimize overfitting by restricting the number of variables included in the multivariable model. Extended treatment was included as the main exposure of interest. Age and sex were included a priori as clinically important covariates, and variables with p < 0.05 in the univariable analysis were additionally considered for inclusion in the multivariable model.

We also agree that diabetes mellitus and chronic pulmonary disease warranted additional evaluation because they showed possible associations with recurrence in the univariable analysis, although they did not meet the predefined p < 0.05 criterion for inclusion in the primary multivariable model. Therefore, we performed additional sensitivity analyses using alternative model specifications that included diabetes mellitus and/or chronic pulmonary disease. The direction of the association between extended treatment and recurrence remained consistent across these models, although the estimates remained imprecise because of the small number of recurrence events. These results are now presented in Supplementary Table S6, and the corresponding description has been added to the Results section (lines 310–315).

**5. Definition of Comorbidities**

**The operational definitions for comorbid conditions are incompletely described. Only chronic pulmonary disease is explicitly defined. For other comorbidities—particularly diabetes mellitus, hypertension, chronic kidney disease, and chronic liver disease—it is unclear whether the diagnoses were based on patient self-report, diagnostic codes, or predefined laboratory criteria (e.g., HbA1c thresholds, eGFR cutoffs). This is especially relevant for diabetes mellitus, which showed a near-significant association with recurrence. The authors should provide clear operational definitions for all comorbid conditions in the Methods section.**

Thank you very much for this helpful and important comment. We agree that the operational definitions of comorbid conditions should be described more clearly.

In response, we have revised the “Data collection” subsection of the Methods section to provide clearer definitions of the comorbidities assessed in this study. We now clarify that comorbidities were defined based on documented diagnoses, relevant medication use, or available clinical information in the medical records. We also added brief operational definitions for diabetes mellitus, hypertension, chronic kidney disease, chronic liver disease, cardiovascular disease, neurologic disease, malignancy, and chronic pulmonary disease. These revisions have been added to the “Data collection” subsection of the Methods section.

**6. Definition of Adverse Drug Reactions**

**Table 3 reports multiple categories of adverse drug reactions including hepatotoxicity, nephrotoxicity, ototoxicity, and cutaneous reactions, but no diagnostic criteria or grading systems are provided in the Methods. Only leukopenia is defined (WBC < 4,000 cells/µL) in a table footnote. Since adverse events represent a key secondary outcome of this study, standardized definitions or grading criteria (e.g., CTCAE grades, specific AST/ALT thresholds for hepatotoxicity) should be provided to ensure reproducibility and allow comparison with existing literature.**

Thank you very much for this helpful and important comment. We agree that the adverse drug reaction categories should be defined more clearly because adverse events were an important secondary safety outcome. In response, we revised the ‘Data collection’ subsection of the Methods section to clarify that adverse drug reactions were identified from the cohort medical records and categorized using operational definitions adapted from CTCAE version 5.0 where applicable. We also clarified that formal CTCAE severity grades were not uniformly recorded in the cohort database; therefore, adverse drug reactions were analyzed as binary outcomes based on their presence or absence rather than by severity grade.

In addition, we added detailed operational definitions for each adverse drug reaction category in the footnote to Table 3, including gastrointestinal adverse reactions, hepatotoxicity, cutaneous adverse drug reactions, leukopenia, generalized weakness, ototoxicity, nephrotoxicity, and infection. (Table 3)

**7. Missing Baseline Laboratory Data**

**The Methods section states that biochemical tests including complete blood count, liver function tests, renal function tests, and inflammation markers were collected at baseline. However, none of these results are presented in the baseline comparison (Table 1). These variables may serve as important confounders that differ between groups and could influence both the clinical decision to extend treatment and the risk of recurrence. It would be beneficial to present these data in the manuscript or supplementary materials, or to explain why they were omitted.**

Thank you very much for this insightful comment. We agree that baseline laboratory findings may represent clinically relevant confounders, as they could influence both the decision to extend treatment and subsequent treatment outcomes.

In response, we reviewed the available baseline laboratory variables in the analytic dataset and added a new supplementary table comparing baseline laboratory abnormalities between the standard- and extended-treatment groups. Specifically, leukopenia, anemia, thrombocytopenia, abnormal liver function, and abnormal renal function are now presented in S1 Table, with footnotes providing the operational definition of each variable. We also added a brief description of these findings to the Results section. (lines 244-248)

In addition, these baseline laboratory abnormalities were evaluated in the univariable logistic regression analysis presented in Supporting Table S3, and none showed a statistically significant association with recurrence. Given the absence of statistically significant univariable associations and the very small number of recurrence events, we did not include these variables in the multivariable model, in order to avoid overfitting and unstable estimates. We also added footnotes to Supporting Table S3 to clarify the operational definitions of the laboratory variables.

**8. Missing Data for 2-Month Culture Results**

**Two-month culture results were available for only 77 of 159 patients (48.4%). This is an important limitation, as the combination of baseline cavity and 2-month culture positivity is the guideline-defined indication for treatment extension. The high proportion of missing data limits the ability to evaluate this clinically relevant subgroup. The authors should discuss whether the missingness was random or systematic, and consider sensitivity analyses that account for missing data.**

Thank you very much for this important comment. We agree that the incomplete availability of two-month culture results limits the evaluation of the clinically relevant subgroup defined by baseline cavitation and persistent culture positivity at 2 months.

As also noted in our response to Reviewer 1 regarding sputum collection intervals, sputum smear and culture examinations were intended to be performed according to the cohort protocol, including assessment at approximately 2 months after treatment initiation. However, because this was a real-world multicenter cohort, two-month culture results were not available for all patients. This may have reflected practical factors encountered during routine care, including difficulty obtaining sputum after clinical improvement, variation in visit timing around the two-month assessment window, clinician discretion regarding follow-up testing, differences in institutional practice patterns, and incomplete capture of test results in the cohort database.

In response, we further examined the pattern of missing two-month culture data. Two-month culture results were missing in 26 of 58 patients (44.8%) in the standard-treatment group and 56 of 101 patients (55.4%) in the extended-treatment group, with no statistically significant between-group difference. We also added Supporting Table S7, which presents sensitivity analyses treating missing two-month culture results as either negative or positive. In these analyses, the between-group difference in two-month culture positivity remained statistically nonsignificant. Univariable logistic analyses also showed no significant association between two-month culture positivity and recurrence under these assumptions.

Nevertheless, we agree that these analyses cannot fully overcome the limitation caused by the high proportion of missing two-month culture data. Therefore, we expanded the ‘3^rd^ Limitation’ in disccusion section to clarify that the prognostic importance of two-month culture positivity, as well as the treatment effect in the guideline-defined subgroup of patients with both baseline cavitation and persistent culture positivity at 2 months, could not be reliably assessed.

**9. Analytical Approach and the 200-Day Threshold**

**While the rationale for using 200 days rather than 180 days as the threshold is explained, the choice of a fixed-time logistic regression approach rather than time-to-event analysis (e.g., Cox proportional hazards model) or modeling treatment duration as a continuous variable warrants further justification. The Discussion mentions these alternatives as future directions, but a brief explanation of why they were not applied in the current analysis would strengthen the methodological transparency.**

Thank you for this helpful comment. We agree that the analytic approach requires clearer justification. We have revised the Statistical Analysis section to explain why a fixed-time logistic regression approach was selected and why time-to-event or continuous-duration modeling was not applied as the primary analysis.

Specifically, because total treatment duration was determined during treatment and was fully known only after treatment completion, treating extended treatment as a baseline exposure in a conventional Cox proportional hazards model could introduce immortal-time bias. Although a time-dependent Cox model could theoretically address this issue, it was not suitable for the present dataset because only four bacteriologically confirmed recurrence events occurred. Similarly, modeling treatment duration as a continuous variable would have required a more complex model and would have produced highly unstable estimates given the very small number of events. Therefore, we used a fixed-time 200-day exposure definition and Firth’s penalized logistic regression, which is more appropriate for sparse outcome data and separation. ( ‘Stastical analysis’ subsection of Methods section)

We also clarified this limitation in the Discussion by noting that time-dependent Cox modeling or continuous treatment-duration modeling could not be reliably applied in the present study because these approaches would have been highly unstable with only four recurrence events. (Second limitation paragraph: lines 390-393)

**10. Tone of Conclusions**

**The conclusion states that “cavity on chest radiograph alone may not be sufficient to justify routine treatment extension beyond 200 days.” Given that only 4 recurrence events were observed and the adjusted analysis showed a trend favoring extended treatment (aOR 0.07, p=0.06), this statement appears to extend beyond what the data can reliably support. A more measured conclusion—such as “this study was underpowered to definitively determine the benefit of routine treatment extension in patients with cavitary disease”—would better reflect the limitations of the evidence.**

Thank you for this important comment. We agree that the original conclusion could be interpreted as more definitive than warranted, particularly given the very small number of recurrence events and the imprecision of the adjusted estimate. Consistent with our response to Reviewer 1, Comment 1, we have revised the conclusion to more clearly state that the study was underpowered to definitively determine the benefit of treatment extension beyond 200 days. We also removed language that could imply absence of benefit and instead emphasized that the findings should be interpreted as exploratory and require confirmation in larger prospective studies.

Once again, I appreciate the authors’ efforts in conducting this multicenter study on a clinically important topic. If the above comments are adequately addressed, I believe the manuscript will provide a meaningful contribution to the evidence base regarding treatment duration in cavitary pulmonary tuberculosis.

Best regards.
