# Supplementary material for: Extended treatment duration and recurrence in successfully treated drug-susceptible pulmonary tuberculosis with cavity on chest radiograph
Source: PLoS One. 2026 Sep 10;21(9):e0357772. doi: 10.1371/journal.pone.0357772 (PMC13561312; doi:10.1371/journal.pone.0357772)
Supplement: Supplementary file 9 [file pone.0357772.s010.docx]

We sincerely thank the Editor and Reviewers for their thoughtful and constructive feedback on our manuscript. We are grateful for the opportunity to revise the manuscript further. We have carefully considered all comments and revised the manuscript accordingly to improve its clarity, consistency, and transparency. Below, we provide a point-by-point response to the Editor’s and Reviewers’ comments. All changes have been incorporated into the revised manuscript and are shown in the tracked-changes version.

**Additional Editor Comments:**

**The multivariable Firth penalized logistic regression should be presented more explicitly as an exploratory analysis. Although Firth’s method is appropriate for sparse data and separation, it cannot overcome the fundamental limitation imposed by only four recurrence events.**

Thank you for this insightful comment. We agree that the multivariable Firth penalized logistic regression should be presented more explicitly as exploratory, given the very small number of recurrence events. We revised the wording throughout the manuscript, including the Abstract conclusion, Results, and Discussion, to clarify that the adjusted analysis was exploratory and that the findings should not be interpreted as definitive evidence.

**Moreover, because BMI was missing in 23 patients, the adjusted model appears to have been based on a complete-case sample and potentially fewer than four recurrence events. Please clearly report how missing BMI data were handled, as well as the exact number of patients and recurrence events included in the multivariable model, preferably in a footnote to Table 4.**

We also appreciate this important point regarding the handling of missing BMI data. Because BMI data were missing in 23 patients, the multivariable model including BMI was conducted as a complete-case analysis without imputation. Accordingly, this model included 136 patients and 3 recurrence events; one recurrence case with missing BMI was excluded. To make this information clear and transparent for readers, we added a footnote to Table 4 specifying the analytic sample size and the number of recurrence events included in the multivariable model.

(Table 4, Lines 319-321)

**The adjusted estimates should not be described as demonstrating independent effects or model stability. Instead, they should be framed as highly imprecise, hypothesis-generating associations. The terms “primary analysis,” “independent predictor,” and “independent determinant” should therefore be replaced with more cautious wording such as “exploratory adjusted analysis” and “association with recurrence.”**

Thank you for these insightful comments. First, in the Statistical Analysis subsection of the Methods, we revised the wording in the sentences explaining why Cox proportional hazards modeling and time-dependent or continuous treatment-duration modeling were not applied. Specifically, we replaced the term “primary analysis” with “the main analytic approach” to avoid implying a definitive primary causal framework.

(‘Statistical analysis’ subsection of Method, lines 206,210)

In the Results section, we also replaced “primary analysis” with “exploratory adjusted analysis” when describing the multivariable Firth penalized logistic regression results, to more clearly reflect the exploratory nature of this analysis.

(Result section, line 307)

In addition, we revised the Discussion paragraph on low BMI by replacing phrasing that could imply low BMI was an “independent” determinant with more cautious language, stating that nutritional status may be clinically relevant when considering long-term treatment outcomes. We then linked this statement to the caution that interpretation is needed because the finding was based on very few recurrence events and had a wide confidence interval.

(Discussion section, lines 382-384)

In addition, because wording related to “stability” in the sensitivity analysis section could be misinterpreted as implying model stability, we revised the sentence to state more cautiously that analyses using alternative model specifications with varying degrees of covariate adjustment showed a similar direction of association.

(Result section, lines 328-329)

**The Abstract should also avoid emphasizing the extreme adjusted OR for low BMI without clearly stating its substantial imprecision.**

We also revised the Abstract to avoid emphasizing the extreme adjusted OR for low BMI. Specifically, we removed the numerical adjusted OR and confidence interval for low BMI from the Abstract and instead stated that low BMI was associated with recurrence, although the estimate was highly imprecise.

( Result of Abstract, lines 100-101)

**Reviewer #1:** I thank the authors for their thorough and, in several places, commendably candid response to the previous round of review. The revision meaningfully improves the manuscript: the title now reflects the analytic population, the tone of the Discussion conclusion is appropriately hedged, operational definitions for comorbidities and adverse drug reactions have been added, baseline laboratory data and missing-data patterns are now reported, and useful sensitivity analyses have been supplied.
The fundamental constraint of this study ("four recurrence events") cannot be resolved by any analytic method, and the authors now reasonably frame their findings as exploratory. My remaining concerns are therefore not requests for new analyses, but requests that the reporting be made fully consistent with that exploratory framing and fully transparent about what the primary model actually estimates. Several of these should be addressed before the manuscript is suitable for publication.

**Major points
1. The Abstract conclusion remains overstated and is now inconsistent with the revised Discussion. In the response letter and in the Discussion (final paragraph), the authors state that they removed language suggesting a benefit in selected patients and reframed the findings as exploratory and imprecise. However, the Abstract conclusion still reads that "our findings indicate a potential benefit of extended treatment in selected patients, warranting prospective evaluation of risk-stratified extension strategies." As the Abstract is what most readers will see, please revise it to match the hedged wording of the Discussion conclusion.**
 We thank the reviewer for this important observation and agree that the Abstract conclusion was inconsistent with the revised Discussion. We have revised the Abstract conclusion to align with the hedged, exploratory framing used in the Discussion, while retaining the study's core clinical message (i.e., that cavity alone does not justify routine extension) so that the Abstract continues to convey the paper's principal contribution rather than reading as a mere restatement of its limitations. We have also revised the wording so that the reference to microbiologic response and disease burden explicitly refers to variables that future studies should systematically capture, consistent with our Limitations section noting that detailed cavitation-specific variables (cavity size, number, extent) were not recorded in the present cohort and that two-month culture data had substantial missingness.

The revised Abstract conclusion now reads:

“Cavity on chest radiograph alone may not be sufficient to justify routine treatment extension beyond 200 days after treatment success. Because only four recurrence events were observed, the adjusted association favoring extended treatment was imprecise and should be interpreted as exploratory rather than definitive. Larger prospective studies that systematically capture microbiologic response and detailed radiologic disease burden are needed to identify which patients with cavitary disease may benefit from individualized treatment extension”

**2. The extreme low-BMI estimate is presented in the Abstract without any indication of imprecision. The Abstract reports an adjusted OR of 81.92 (95% CI, 4.95–21,694.24). This estimate arises from near-complete separation (0/110 vs. 3/26) and its magnitude is not interpretable. Consistent with the caveat the authors added to the Discussion, I recommend removing the numeric estimate from the Abstract and instead stating that low BMI was associated with recurrence but that the estimate was very imprecise.**
 Thank you for this insightful comment. We agree that presenting the numerical low-BMI estimate in the Abstract may overemphasize an unstable estimate based on very few recurrence events. Accordingly, we removed the adjusted OR and confidence interval from the Abstract and revised the sentence to state that low BMI was associated with recurrence, while emphasizing that the estimate was highly imprecise.

The revised sentence in the Abstract Results now reads:

"Among covariates, low body mass index was associated with recurrence, but this estimate was very imprecise and should be interpreted with caution."

In addition, consistent with this revision, we also modified the wording in the Discussion regarding low BMI. Specifically, we replaced phrasing that could imply low BMI was an “independent” determinant with more cautious language, stating that nutritional status may be clinically relevant when considering long-term treatment outcomes. We then linked this statement to the caution that interpretation is needed because the finding was based on very few recurrence events and had a wide confidence interval.

The revised sentence in the Discussion section now reads: “Our results are therefore concordant with existing evidence and suggest that nutritional status may be clinically relevant to long-term treatment outcomes.”

Furthermore, although the numeric estimate was retained in the Results section of the main text for completeness and transparency, we revised that sentence to explicitly flag its imprecision, so that readers encountering the estimate outside the Abstract are not misled by its magnitude. The revised sentence in the Results section now reads: "In contrast, low body mass index (BMI) (<18.5 kg/m²) was associated with recurrence in the multivariable Firth penalized logistic regression model (OR, 81.92; 95% CI, 4.95–21,694.24; p < 0.001), although this estimate was highly imprecise due to sparse data (see Table 4 footnote)."

**3. The analytic sample size and number of events contributing to the multivariable model must be reported. The primary multivariable model includes BMI, which is missing in 23 patients (14.5%), and no imputation is described, implying a complete-case analysis of approximately 136 patients. Because three of the four recurrence events occurred in the low-BMI group and the fourth appears to fall among the BMI-missing patients, the multivariable model may be based on as few as three events. Please state explicitly (a) how missing BMI was handled, and (b) the actual N and number of recurrence events contributing to the multivariable model, ideally in a footnote to Table 4. This information is essential for readers to judge the primary estimate.**

Thank you for this important and insightful comment. We agree that explicitly reporting the analytic sample size and the number of recurrence events contributing to the multivariable model would improve the transparency and interpretability of the results. Because BMI data were missing in 23 patients, the primary multivariable Firth penalized logistic regression model, which included BMI, was conducted as a complete-case analysis without imputation. Accordingly, this model included 136 patients and 3 recurrence events; one recurrence case with missing BMI was excluded. To make this clearer for readers, we added a footnote to Table 4 specifying the actual analytic sample size and event count used in the multivariable model. (Result section, Table 4 footnote, lines 319-321).

In addition, in Table 4, we revised confidence interval lower bounds that had been rounded to 0.00 and reported them as <0.01 to avoid misinterpretation. (Table 4)

**Minor points**
**4. Given the numeric corrections required in this revision, I would ask the authors to independently re-verify all figures in the final version. Specific inconsistencies I noted:
- The Discussion reports baseline AFB smear positivity in the standard group as 30.2%, whereas Table 1, the Abstract, and the Results state 29.3%. Please reconcile.**
 Thank you for pointing out this inconsistency. We have corrected the baseline AFB smear positivity in the Discussion from 30.2% to 29.3% for the standard-treatment group, so that it is consistent with Table 1, the Abstract, and the Results section.

(Discussion section, lines 358)

**- The coordinating-institution IRB number appears as "C19ONDI0458" in the Ethics Statement but as "KC19ONDI0458" elsewhere. Please use a single consistent number.**
 Thank you for carefully noting this inconsistency. After rechecking the coordinating-institution IRB approval number, we standardized it as “KC19ONDI0458” throughout the manuscript to ensure consistency.

(Methods section, lines 227)

**- Two-month culture positivity is reported as 5.1% in the Discussion but 5.2% in Table 2; please standardize the rounding.**

Thank you for carefully noting this inconsistency. We have standardized the reported proportion of two-month culture positivity as 5.2% in the Discussion, consistent with the value presented in Table 2.

(Discussion section, lines 419)

**5. Supplementary materials. Please confirm that the final Supporting Information file is complete and that its table numbering matches the in-text citations (S1–S7), including the recurrence-case summary and the sensitivity analyses added in this revision. The numbering of the supplementary tables and the in-text references should be checked against one another.**

Thank you for this helpful comment. We carefully reviewed the final Supporting Information file and cross-checked all supplementary table numbers against the corresponding in-text citations. We confirmed that the Supporting Information file includes Supporting Tables S1–S7, including the recurrence-case summary and the sensitivity analyses added during revision, and that the numbering of the supplementary tables is consistent with the in-text references.

**Reviewer #2:** Dear Authors,
Thank you for your thorough and thoughtful revision. The manuscript has been substantially improved, and I appreciate the considerable effort taken to address each of my previous comments. The revised title now accurately reflects the study population, the survivorship bias and confounding by indication are explicitly discussed, comorbidity and adverse drug reaction definitions have been added, baseline laboratory data and missing data analyses are now provided, and the Discussion conclusion has been appropriately tempered. The addition of a sample size estimation for future studies and the clinical description of the four recurrence cases are also valuable.
I have only two remaining concerns, both of which should be straightforward to address.

**1. Inconsistency Between the Abstract Conclusion and the Discussion Conclusion
The Discussion conclusion has been revised to state that the study was underpowered and that findings should be interpreted as exploratory, which I believe is appropriate. However, the Abstract conclusion still retains the original wording: “Cavity on chest radiograph alone may not be sufficient to justify routine treatment extension beyond 200 days after treatment success. Nevertheless, our findings indicate a potential benefit of extended treatment in selected patients.” This language is more assertive than what the Discussion now conveys and creates an internal inconsistency within the manuscript. Because the Abstract is often the only section read by many clinicians, it is important that its tone matches the more measured Discussion conclusion. I would suggest revising the Abstract conclusion to align with the revised Discussion.**

Thank you for this important and helpful comment. We fully agree that the Abstract conclusion should be consistent with the more cautious wording of the Discussion conclusion, particularly given the limited number of recurrence events. Accordingly, we have revised the Abstract conclusion to more clearly emphasize the small number of recurrence events, the imprecision of the adjusted estimate, and the exploratory nature of the findings.

The revised Abstract conclusion now reads:

“Cavity on chest radiograph alone may not be sufficient to justify routine treatment extension beyond 200 days after treatment success. Because only four recurrence events were observed, the adjusted association favoring extended treatment was imprecise and should be interpreted as exploratory rather than definitive. Larger prospective studies that systematically capture microbiologic response and detailed radiologic disease burden are needed to identify which patients with cavitary disease may benefit from individualized treatment extension”

**2. Residual Use of “Independent Determinant” in the Discussion
In the Discussion paragraph on low BMI (line 378), the phrase “independent determinant of long-term treatment outcomes” remains. While the cautionary sentence that follows is appreciated, the term “independent determinant” still implies a level of certainty that is not supported by an estimate based on four events with a confidence interval spanning four orders of magnitude. Replacing this with more cautious language, consistent with the revisions already made elsewhere in the manuscript, would ensure internal consistency.**

Thank you for this thoughtful comment. We agree that the term “independent determinant” was too strong in light of the sparse number of recurrence events and the very wide confidence interval. We therefore revised the relevant Discussion sentence to avoid implying a definitive independent effect of low BMI. The revised wording now describes nutritional status as potentially clinically relevant to long-term treatment outcomes and maintains a cautious interpretation of this finding.

The revised sentence in the Discussion section now reads:

“Our results are therefore concordant with existing evidence and suggest that nutritional status may be clinically relevant to long-term treatment outcomes.”

We again sincerely thank the Editor and Reviewers for their careful evaluation of our manuscript and for the constructive comments that have helped us improve its clarity, transparency, and interpretability. We hope that the revised manuscript and our responses adequately address all remaining concerns.
