# Supplementary material for: Extended treatment duration and recurrence in successfully treated drug-susceptible pulmonary tuberculosis with cavity on chest radiograph
Source: PLoS One. 2026 Sep 10;21(9):e0357772. doi: 10.1371/journal.pone.0357772 (PMC13561312; doi:10.1371/journal.pone.0357772)
Supplement: Peer review history

# Peer review: Decision Letter 0

**DOI:** 10.1371/journal.pone.0357772.r001

**Authors:** Jung Yeon Heo

6 May 2026

Dear Dr. Kwon,

Thank you for submitting your manuscript to PLOS ONE. After careful consideration, we feel that it has merit but does not fully meet PLOS ONE’s publication criteria as it currently stands. Therefore, we invite you to submit a revised version of the manuscript that addresses the points raised during the review process.

plosone@plos.org. When you're ready to submit your revision, log on to https://www.editorialmanager.com/pone/ and select the 'Submissions Needing Revision' folder to locate your manuscript file.

- A letter that responds to each point raised by the academic editor and reviewer(s). You should upload this letter as a separate file labeled 'Response to Reviewers'.
- A marked-up copy of your manuscript that highlights changes made to the original version. You should upload this as a separate file labeled 'Revised Manuscript with Track Changes'.
- An unmarked version of your revised paper without tracked changes. You should upload this as a separate file labeled 'Manuscript'.

If you would like to make changes to your financial disclosure, please include your updated statement in your cover letter. Guidelines for resubmitting your figure files are available below the reviewer comments at the end of this letter.

If applicable, we recommend that you deposit your laboratory protocols in protocols.io to enhance the reproducibility of your results. Protocols.io assigns your protocol its own identifier (DOI) so that it can be cited independently in the future. For instructions see: https://journals.plos.org/plosone/s/submission-guidelines#loc-laboratory-protocols. Additionally, PLOS ONE offers an option for publishing peer-reviewed Lab Protocol articles, which describe protocols hosted on protocols.io. Read more information on sharing protocols at https://plos.org/protocols?utm_medium=editorial-email&utm_source=authorletters&utm_campaign=protocols.

As the corresponding author, your ORCID iD is verified in the submission system and will appear in the published article. PLOS supports the use of ORCID, and we encourage all coauthors to register for an ORCID iD and use it as well. Please encourage your coauthors to verify their ORCID iD within the submission system before final acceptance, as unverified ORCID iDs will not appear in the published article. *Only*  the individual author can complete the verification step; PLOS staff *cannot*  verify ORCID iDs on behalf of authors.

We look forward to receiving your revised manuscript.

Kind regards,

Jung Yeon Heo

Academic Editor

PLOS One

**Journal Requirements:**

When submitting your revision, we need you to address these additional requirements.

1. Please ensure that your manuscript meets PLOS ONE's style requirements, including those for file naming. The PLOS ONE style templates can be found at

https://journals.plos.org/plosone/s/file?id=wjVg/PLOSOne_formatting_sample_main_body.pdf and

https://journals.plos.org/plosone/s/file?id=ba62/PLOSOne_formatting_sample_title_authors_affiliations.pdf

2. Thank you for stating the following financial disclosure:

“This work was supported by the Research Program funded by a grant (BCRI24033) from Chonnam National University Hospital Biomedical Research Institute.”

Please state what role the funders took in the study.  If the funders had no role, please state: "The funders had no role in study design, data collection and analysis, decision to publish, or preparation of the manuscript."

If this statement is not correct you must amend it as needed.

Please include this amended Role of Funder statement in your cover letter; we will change the online submission form on your behalf.

3. Please note that funding information should not appear in any section or other areas of your manuscript. We will only publish funding information present in the Funding Statement section of the online submission form. Please remove any funding-related text from the manuscript.

4. You have indicated that data is available from [ whitevet81@korea.kr]. Please can we ask you to provide us with a general contact email address for the data requests, so readers can request access in perpetuity. If a general email is not available please provide a link to a website where readers can obtain access to data.

5. In the online submission form you indicate that your data is not available for proprietary reasons and have provided a contact point for accessing this data. Please note that your current contact point is a co-author on this manuscript. According to our Data Policy, the contact point must not be an author on the manuscript and must be an institutional contact, ideally not an individual. Please revise your data statement to a non-author institutional point of contact, such as a data access or ethics committee, and send this to us via return email. Please also include contact information for the third party organization, and please include the full citation of where the data can be found.

6. Please include captions for your Supporting Information files at the end of your manuscript, and update any in-text citations to match accordingly. Please see our Supporting Information guidelines for more information: http://journals.plos.org/plosone/s/supporting-information.

7. If the reviewer comments include a recommendation to cite specific previously published works, please review and evaluate these publications to determine whether they are relevant and should be cited. There is no requirement to cite these works unless the editor has indicated otherwise.

**Additional Editor Comments :**

Thank you for your submission to PLOS ONE. I apologize for the delay in issuing this decision due to my clinical and academic commitments.

The manuscript was reviewed by two reviewers, both of whom provided detailed and constructive comments. After considering their evaluations, the decision is major revision.

Please revise the manuscript accordingly and submit a detailed point-by-point response to all comments. A careful and thorough revision will be important for further consideration of the manuscript.

[Note: HTML markup is below. Please do not edit.]

Reviewers' comments:

Reviewer's Responses to Questions

**Comments to the Author**

1. Is the manuscript technically sound, and do the data support the conclusions?

Reviewer #1: Partly

Reviewer #2: Yes

**********

2. Has the statistical analysis been performed appropriately and rigorously?

Reviewer #1: Yes

Reviewer #2: Yes

**********

3. Have the authors made all data underlying the findings in their manuscript fully available?

The PLOS Data policy

Reviewer #1: Yes

Reviewer #2: No

**********

4. Is the manuscript presented in an intelligible fashion and written in standard English?

Reviewer #1: Yes

Reviewer #2: Yes

**********

Reviewer #1: Major Comments

1. Underpowered study and overstated conclusions

The primary analysis yielded an aOR of 0.07 (95% CI 0.01–1.06, p = 0.06), based on only four recurrence events, and did not reach statistical significance. Describing this as "a protective trend" risks overstating the evidence. The conclusion should more explicitly acknowledge that this study was underpowered to draw definitive conclusions. A post-hoc power calculation or sample size estimation for a future adequately powered study should be provided.

2. Absence of predefined indications for treatment extension

No protocol-defined criteria for extending treatment are described, and the Discussion acknowledges that extension was largely driven by clinician judgment. If the clinical reasons for treatment extension can be retrieved retrospectively, they should be reported. If not, this should be explicitly acknowledged as a limitation.

3. Radiologic severity of cavitation not captured

Cavity size, number, and extent of pulmonary involvement are recognized prognostic factors yet were not incorporated into the analysis. The authors should explore whether these data were recorded and, if so, include them in the analysis. If unavailable, this should be discussed more prominently as a source of residual confounding.

4. Missing BMI data

BMI was the strongest independent predictor of recurrence, yet data were available for only 136 of 159 patients (85.5%). The reasons for and distribution of missing BMI data across groups should be reported, and the potential impact on model estimates should be addressed.

5. IRB approval for all participating institutions

Only a single IRB approval number is provided for an 18-center study. The authors should confirm that central IRB approval covered all sites or provide individual approval numbers for each institution.

6. Sputum collection intervals

The timing and frequency of sputum collection are not described. Given that two-month culture positivity and culture conversion are key outcomes, and the number of patients with available data differed between groups (n = 32 vs. 45), the authors should confirm that sputum collection intervals were comparable between groups.

Minor Comments

1. The abstract should include a brief description of the source cohort.

2. Please explicitly state that only patients susceptible to all HRZE components were included, using the term "pan-susceptible" or equivalent, to avoid confusion with the current sequential exclusion criteria.

3. Specific patient numbers in the exclusion process should be moved from the Methods to the Results section or flow diagram legend.

4. "Gender" should be replaced with "sex" throughout the manuscript, consistent with ICMJE recommendations.

5. The rationale for the 200-day cutoff should be more explicitly justified in the Methods, with a clearer explanation of how the referenced study (ref 15) informed this threshold.

6. The average duration of follow-up after treatment completion and its comparability between groups should be reported.

7. P-values in Supplementary Table 2 should be reported to at least three decimal places.

8. A brief clinical description of the four recurrence cases (timing, group assignment, suspected relapse vs. reinfection) would provide useful context for interpreting the primary outcome.

Reviewer #2: Dear Authors,

I appreciate the effort that went into this multicenter study evaluating the potential benefit of extended treatment in drug-susceptible pulmonary tuberculosis patients with cavitary disease.

The study addresses a clinically relevant and timely question, given the limited evidence base supporting current guideline recommendations for treatment extension.

The utilization of a well-established prospective cohort from 18 Korean institutions is a notable strength, and the manuscript is clearly written with an appropriate structure and length.

However, there are several methodological and interpretive aspects that require further clarification and discussion before the manuscript can be considered for publication. I have outlined my specific comments below.

1. Study Population and Potential Survivorship Bias

The study population is restricted to patients who achieved treatment success with the HRZE regimen, having excluded those who experienced treatment failure (n=4), death (n=53), loss to follow-up (n=8), and transfer to other clinics (n=41). This introduces a significant survivorship bias, as patients who received extended treatment but could not complete therapy due to adverse events or who died during treatment are systematically excluded from the analysis.

Consequently, the study does not evaluate the “efficacy of extended treatment” as the title implies, but rather compares recurrence rates among patients who successfully completed their respective treatment courses. This distinction is important and should be reflected in the title, abstract, and conclusions. Additionally, the Discussion section should include a dedicated paragraph addressing how this selection may have influenced the results.

2. Extremely Low Number of Recurrence Events and Model Stability

With only 4 recurrence events among 159 patients, the multivariable Firth’s penalized logistic regression model including 4 covariates (extended treatment, age, sex, and BMI) yields an events-per-variable ratio below 1.

While the choice of Firth’s method is appropriate to address separation, the fundamental limitation imposed by the extremely low event count cannot be fully overcome by any statistical technique.

This is clearly reflected in the confidence interval for low BMI (OR 81.92; 95% CI, 4.95–21,694.24), which spans over four orders of magnitude.

Characterizing this variable as an independent predictor may overstate the precision and reliability of the estimate.

While the direction of association is biologically plausible and consistent with prior literature, the magnitude should be interpreted with explicit caution.

It would be beneficial to add appropriate caveats both in the Results and Discussion sections.

3. Confounding by Indication

The extended treatment group had significantly higher baseline AFB smear positivity (56.4% vs. 29.3%, p<0.01), suggesting that clinicians selectively extended treatment for patients with perceived greater disease severity.

This introduces confounding by indication—a well-recognized limitation in observational treatment-duration studies.

If the extended treatment group had a higher baseline disease burden yet showed similar recurrence rates to the standard group, this observation could reflect either a genuine protective effect of extended treatment or residual confounding. The current analysis does not adequately address this issue. The authors should consider discussing the potential utility of methods such as propensity score matching or inverse probability of treatment weighting, and at minimum provide a thorough discussion of how confounding by indication may have affected the interpretation of results.

4. Variable Selection in the Multivariable Model

The rationale for variable selection in the multivariable model is not fully transparent.

Age and sex were included a priori, and BMI was included based on clinical relevance and consistency with prior evidence. However, in the univariable analysis, diabetes mellitus (OR 5.49, p=0.07) and chronic pulmonary disease (OR 9.86, p=0.06) demonstrated associations with recurrence that were at least as strong as, or stronger than, several included variables, yet they were not selected for the final model.

Given the severe constraint imposed by the small number of events, it is understandable that not all variables could be included.

Nevertheless, the authors should provide explicit criteria for variable selection and consider presenting alternative model specifications with different covariate combinations in sensitivity analyses to demonstrate the robustness of the findings.

5. Definition of Comorbidities

The operational definitions for comorbid conditions are incompletely described. Only chronic pulmonary disease is explicitly defined. For other comorbidities—particularly diabetes mellitus, hypertension, chronic kidney disease, and chronic liver disease—it is unclear whether the diagnoses were based on patient self-report, diagnostic codes, or predefined laboratory criteria (e.g., HbA1c thresholds, eGFR cutoffs). This is especially relevant for diabetes mellitus, which showed a near-significant association with recurrence. The authors should provide clear operational definitions for all comorbid conditions in the Methods section.

6. Definition of Adverse Drug Reactions

Table 3 reports multiple categories of adverse drug reactions including hepatotoxicity, nephrotoxicity, ototoxicity, and cutaneous reactions, but no diagnostic criteria or grading systems are provided in the Methods. Only leukopenia is defined (WBC < 4,000 cells/µL) in a table footnote. Since adverse events represent a key secondary outcome of this study, standardized definitions or grading criteria (e.g., CTCAE grades, specific AST/ALT thresholds for hepatotoxicity) should be provided to ensure reproducibility and allow comparison with existing literature.

7. Missing Baseline Laboratory Data

The Methods section states that biochemical tests including complete blood count, liver function tests, renal function tests, and inflammation markers were collected at baseline. However, none of these results are presented in the baseline comparison (Table 1). These variables may serve as important confounders that differ between groups and could influence both the clinical decision to extend treatment and the risk of recurrence. It would be beneficial to present these data in the manuscript or supplementary materials, or to explain why they were omitted.

8. Missing Data for 2-Month Culture Results

Two-month culture results were available for only 77 of 159 patients (48.4%). This is an important limitation, as the combination of baseline cavity and 2-month culture positivity is the guideline-defined indication for treatment extension. The high proportion of missing data limits the ability to evaluate this clinically relevant subgroup. The authors should discuss whether the missingness was random or systematic, and consider sensitivity analyses that account for missing data.

9. Analytical Approach and the 200-Day Threshold

While the rationale for using 200 days rather than 180 days as the threshold is explained, the choice of a fixed-time logistic regression approach rather than time-to-event analysis (e.g., Cox proportional hazards model) or modeling treatment duration as a continuous variable warrants further justification. The Discussion mentions these alternatives as future directions, but a brief explanation of why they were not applied in the current analysis would strengthen the methodological transparency.

10. Tone of Conclusions

The conclusion states that “cavity on chest radiograph alone may not be sufficient to justify routine treatment extension beyond 200 days.” Given that only 4 recurrence events were observed and the adjusted analysis showed a trend favoring extended treatment (aOR 0.07, p=0.06), this statement appears to extend beyond what the data can reliably support. A more measured conclusion—such as “this study was underpowered to definitively determine the benefit of routine treatment extension in patients with cavitary disease”—would better reflect the limitations of the evidence.

Once again, I appreciate the authors’ efforts in conducting this multicenter study on a clinically important topic. If the above comments are adequately addressed, I believe the manuscript will provide a meaningful contribution to the evidence base regarding treatment duration in cavitary pulmonary tuberculosis.

Best regards.

**********

what does this mean?). If published, this will include your full peer review and any attached files.

If you choose “no”, your identity will remain anonymous but your review may still be made public.

**Do you want your identity to be public for this peer review?**  For information about this choice, including consent withdrawal, please see our Privacy Policy

Reviewer #1: No

Reviewer #2: No

**********

[NOTE: If reviewer comments were submitted as an attachment file, they will be attached to this email and accessible via the submission site. Please log into your account, locate the manuscript record, and check for the action link "View Attachments". If this link does not appear, there are no attachment files.]

To ensure your figures meet our technical requirements, please review our figure guidelines: https://journals.plos.org/plosone/s/figures

You may also use PLOS’s free figure tool, NAAS, to help you prepare publication quality figures: https://journals.plos.org/plosone/s/figures#loc-tools-for-figure-preparation.

NAAS will assess whether your figures meet our technical requirements by comparing each figure against our figure specifications.

# Author comment: Author response to Decision Letter 1

**DOI:** 10.1371/journal.pone.0357772.r002

3 Jun 2026

Response to the editor and reviewers

Ref: Submission ID PONE-D-26-05397

Title: Efficacy of Extended Treatment in Drug-Susceptible Pulmonary Tuberculosis with Cavity on Chest Radiograph

We sincerely thank the Editor and the Reviewer for their thoughtful and constructive feedback on our manuscript. We have carefully considered and addressed every comment in the revised version of the paper. Below, we present a detailed point-by-point response, with each reviewer’s comment followed by our corresponding reply. All changes have been incorporated into the manuscript, and the tracked-changes document highlights all new or substantially revised sections.

Reviewer #1: Major Comments

1. Underpowered study and overstated conclusions

The primary analysis yielded an aOR of 0.07 (95% CI 0.01–1.06, p = 0.06), based on only four recurrence events, and did not reach statistical significance. Describing this as "a protective trend" risks overstating the evidence. The conclusion should more explicitly acknowledge that this study was underpowered to draw definitive conclusions. A post-hoc power calculation or sample size estimation for a future adequately powered study should be provided.

We thank you for this important comment. We agree that the primary analysis, which was based on only four recurrence events, does not provide sufficient statistical evidence to support a definitive protective effect.

Accordingly, we revised the wording throughout the manuscript to avoid overstating the findings. First, in the Abstract and Results section, we removed the term “trend” from the description of the Firth penalized logistic regression analysis and replaced it with more objective wording (lines 97 and 289–292). Similarly, in the sensitivity analysis, we removed the phrase “protective trend” and revised the text using more objective language (lines 306-307).

In addition, at the beginning of the Discussion, we deleted the sentence containing the phrase “protective trend” to further avoid overstating the results (lines 318–324).

In addition, we have removed the statements in the Results section regarding the benefits of extended treatment to avoid the overstatement you pointed out. Instead, we have emphasized the potential risks of unnecessary treatment extension and its associated adverse effects, and revised the text to highlight the need for careful selection of patients who would require treatment extension in future practice. (Lines 351-355 in result section)

In the Limitations section, we also strengthened the statement that the small sample size limits the generalizability of the findings We replaced wording suggesting cautious interpretation with a clearer statement that the results should be interpreted as exploratory rather than definitive, thereby emphasizing the limited generalizability of the findings (Second paragraph in limitation section).

Furthermore, we added a description of the design considerations for a future study, including a specific sample size estimation, at the end of the second paragraph of the Limitations section.

Finally, consistent with Reviewer 2’s comment on the tone of the conclusion, we revised the concluding statement of the Discussion to avoid overstating our findings. We removed the phrase “suggest a potential benefit in selected patients” and instead emphasized that the study was underpowered, the adjusted estimate was imprecise, and the findings should be interpreted as exploratory. (Conclusion paragraph of the Discussion section)

In addition, as part of our revision and statistical verification process, we carefully re-examined the Firth penalized logistic regression results presented in Table 4. During this review, we noted that the originally calculated univariable estimates for age and sex were not accurately reflected in the previous version of the table. We sincerely apologize for this oversight and have corrected these values in the revised table. Importantly, the multivariable primary model results, including the adjusted estimate for extended treatment, remained unchanged, and these corrections do not affect the overall interpretation of the study findings.

2. Absence of predefined indications for treatment extension

No protocol-defined criteria for extending treatment are described, and the Discussion acknowledges that extension was largely driven by clinician judgment. If the clinical reasons for treatment extension can be retrieved retrospectively, they should be reported. If not, this should be explicitly acknowledged as a limitation.

Thank you for your insightful comment. We agree that treatment extension in our cohort was not governed by predefined protocol-driven criteria but was determined by the clinical judgment of the treating physicians at each participating center. However, the reasons for treatment extension were not documented in the cohort records and therefore could not be reliably retrieved retrospectively.

Accordingly, we have explicitly acknowledged this issue in the fourth limitation paragraph of the revised Discussion. We clarified that the absence of predefined indications for treatment extension and the inability to retrieve the specific rationale for each extension decision may have introduced residual confounding by indication.

3. Radiologic severity of cavitation not captured

Cavity size, number, and extent of pulmonary involvement are recognized prognostic factors yet were not incorporated into the analysis. The authors should explore whether these data were recorded and, if so, include them in the analysis. If unavailable, this should be discussed more prominently as a source of residual confounding.

We thank you for this important comment. As shown in our results, information on multilobar infiltration on chest radiography was available as an indicator of overall disease extent. However, this cohort did not include detailed radiologic data specifically characterizing cavitary disease burden, such as cavity size, number of cavities, or the extent of cavitary involvement. Therefore, these cavitation-specific severity measures could not be reliably retrieved retrospectively or incorporated into the analysis.

Accordingly, we expanded the fifth limitation paragraph of the revised Discussion to address this issue more explicitly. We noted that recent studies have suggested that cavitation-specific features, including cavity size, number of cavities, and extent of cavitary involvement, may serve as indicators of tuberculosis disease burden and recurrence risk, and we additionally cited the relevant supporting references. We also clarified that the lack of detailed radiologic information beyond the binary presence of cavitation may have limited our ability to fully adjust for baseline disease severity.

4. Missing BMI data

BMI was the strongest independent predictor of recurrence, yet data were available for only 136 of 159 patients (85.5%). The reasons for and distribution of missing BMI data across groups should be reported, and the potential impact on model estimates should be addressed.

We thank you for this very insightful comment.

(i) Reasons for missing BMI data:

The missing BMI values were attributable to the absence of height and/or weight information in the cohort records, rather than intentional exclusion. Unfortunately, no additional documentation was available to determine the specific reasons for these missing measurements.

(ii) Distribution of missing data across groups and potential impact on model estimates:

BMI was missing in 14 of 58 patients (24.1%) in the standard-treatment group and in 9 of 101 patients (8.9%) in the extended-treatment group, with a statistically significant difference between groups (p = 0.017). We agree that this imbalance is important, as complete-case analysis may have introduced selection bias. Given that BMI was identified as an important predictor of recurrence, differential missingness in BMI may also have affected the precision and stability of the model estimates.

Accordingly, we have added a new third paragraph to the Limitations section of the revised Discussion. In this paragraph, we describe the imbalance in missing BMI data between treatment groups and explicitly acknowledge that, because BMI was an important predictor of recurrence, this differential missingness may have introduced selection bias and should be considered when interpreting the findings.

5. IRB approval for all participating institutions

Only a single IRB approval number is provided for an 18-center study. The authors should confirm that central IRB approval covered all sites or provide individual approval numbers for each institution.

We thank the reviewer for this important comment. We confirm that the study was conducted in accordance with the Declaration of Helsinki and was approved by the Institutional Review Boards (IRBs) and Ethics Committees of all participating institutions. The study was coordinated and overseen by the representative institution, the Catholic University of Korea (IRB No. C19ONDI0458), which served as the coordinating center for the 17 other participating medical institutions. In accordance with the reviewer's suggestion, we have now provided the individual IRB approval numbers for each participating institution in the revised 'Ethics Statement' subsection of the Methods section, as follows:

The study was conducted in accordance with the Declaration of Helsinki and was approved by the Institutional Review Boards (IRBs) and Ethics Committees of all participating institutions. The study was coordinated and overseen by the representative institution, the Catholic University of Korea (IRB No. KC19ONDI0458), which represented and managed the study across the 17 other participating medical institutions. All participating institutions obtained IRB approval prior to study initiation, with the following approval numbers: SCHBC202012023001-HE001 (Soonchunhyang University Bucheon Hospital), UUH202103016-HE002 (Ulsan University Hospital), GCIRB2021-016 (Gachon University Gil Hospital), 2020GR0402 (Korea University Guro Hospital), VC19ONDI016 (St. Vincent's Hospital), KANGDONG2020-06-012-001 (Kangdong Sacred Heart Hospital), KYUH2020-12-013-002 (Konyang University Hospital), SCHCA202012076-HE005 (Soonchunhyang University Cheonan Hospital), DUIH202101023001-HE001 (Dongguk University Ilsan Hospital), 2020-07-007 (Chungnam National University Hospital), 2020-08-012-003 (Chungbuk National University Hospital), DC19ONDI0052 (Daejeon St. Mary's Hospital), HC19ONDI0068 (Bucheon St. Mary's Hospital), UC19ONDI0106 (Uijeongbu St. Mary's Hospital), PC19ONDI0149 (Eunpyeong St. Mary's Hospital), OC19ONDI0073 (Incheon St. Mary's Hospital), and ISPAIK2020-11-003-001 (Ilsan Paik Hospital). Written informed consent was obtained from all adult participants before their enrolment in the study.

6. Sputum collection intervals

The timing and frequency of sputum collection are not described. Given that two-month culture positivity and culture conversion are key outcomes, and the number of patients with available data differed between groups (n = 32 vs. 45), the authors should confirm that sputum collection intervals were comparable between groups.

We sincerely thank you for this insightful and important comment. We fully agree that the timing and frequency of sputum collection are important considerations when interpreting two-month culture positivity and culture conversion outcomes. According to the cohort protocol, sputum smear and culture examinations were intended to be performed as part of routine follow-up during anti-tuberculosis treatment, including assessment at approximately 2 months after treatment initiation. Therefore, the intended sputum collection schedule was comparable between the standard- and extended-treatment groups.

Nevertheless, two-month culture results were not available for all patients in this real-world multicenter cohort, despite the intended protocol-based follow-up schedule. This incomplete availability may have reflected several factors encountered in routine clinical practice, including inability to expectorate sputum after clinical improvement, variation in visit timing around the two-month assessment window, clinician discretion regarding follow-up testing, differences in institutional practice patterns, or incomplete capture of test results in the cohort records.

In response to this comment, and in addition to the issue of missing BMI data addressed in Comment 4, we further expanded the third limitation paragraph of the revised Discussion to address missing data for clinically important variables more broadly. Specifically, we acknowledged that the incomplete availability of two-month culture data may have limited our ability to fully evaluate the prognostic importance of two-month culture positivity and its association with recurrence.

Minor Comments

1. The abstract should include a brief description of the source cohort.

We thank you for this helpful comment. We have revised the Abstract to include a clearer description of the source cohort. Specifically, we clarified that this study was a retrospective analysis using data from a multicenter prospective cohort of patients with pulmonary tuberculosis enrolled at 18 Korean institutions, and that the present analysis focused on patients with drug-susceptible pulmonary tuberculosis and cavity on chest radiograph.

2. Please explicitly state that only patients susceptible to all HRZE components were included, using the term “pan-susceptible” or equivalent, to avoid confusion with the current sequential exclusion criteria.

Thank you for this helpful suggestion. To clarify the eligibility criteria and avoid confusion with the sequential exclusion criteria, we revised the Methods section to explicitly state that only patients with pan-susceptible tuberculosis were included. Specifically, we added that the study population was restricted to patients susceptible to all HRZE components (‘population’ subsection of Methods section)

3. Specific patient numbers in the exclusion process should be moved from the Methods to the Results section or flow diagram legend.

Thank you very much for this helpful and thoughtful suggestion. We agree that presenting the specific patient numbers for each exclusion step in the Results section or flow diagram is clearer and more appropriate than including them in the Methods section. Accordingly, we have revised the Methods section to describe the eligibility and exclusion criteria without specific patient numbers, and have moved the detailed exclusion counts to the Results section, with reference to Figure 1.

(‘Population’ Subsection of Method section)

(Result section: lines 226-234)

4. "Gender" should be replaced with "sex" throughout the manuscript, consistent with ICMJE recommendations.

Thank you very much for this important suggestion. We have revised the manuscript to replace “gender” with “sex” throughout, in accordance with ICMJE recommendations. (line 153)

5. The rationale for the 200-day cutoff should be more explicitly justified in the Methods, with a clearer explanation of how the referenced study (ref 15) informed this threshold.

Thank you very much for this valuable suggestion. We agree that the rationale for the 200-day cutoff should be more explicitly described to improve the clarity and reproducibility of our Methods. Accordingly, we have revised the Methods section to clarify that the cutoff was based on the standard 6-month regimen, corresponding to approximately 180 days, with an additional 2-week allowance. We also clarified that this allowance was informed by a previous study of prolonged TB treatment [15], which noted that treatment interruptions of ≥2 weeks during the intensive phase may require reintroduction of intensive-phase treatment and thereby prolong the total treatment duration. Thus, the 200-day cutoff was selected to distinguish meaningful treatment extension from minor variations around the standard 6-month course. (‘Group and study outcome’ subsection of Methods section: lines 182-189)

6. The average duration of follow-up after treatment completion and its comparability between groups should be reported.

Thank you very much for this helpful suggestion. We agree that reporting the follow-up

Attachment Submitted filename: Response to review.docx

# Peer review: Decision Letter 1

**DOI:** 10.1371/journal.pone.0357772.r003

**Authors:** Jung Yeon Heo

12 Jul 2026

Dear Dr. Kwon,

Thank you for submitting your manuscript to PLOS ONE. After careful consideration, we feel that it has merit but does not fully meet PLOS ONE’s publication criteria as it currently stands. Therefore, we invite you to submit a revised version of the manuscript that addresses the points raised during the review process.

Please submit your revised manuscript by Aug 26 2026 11:59PM. If you will need more time than this to complete your revisions, please reply to this message or contact the journal office at plosone@plos.org. When you're ready to submit your revision, log on to https://www.editorialmanager.com/pone/ and select the 'Submissions Needing Revision' folder to locate your manuscript file.

- A letter that responds to each point raised by the academic editor and reviewer(s). You should upload this letter as a separate file labeled 'Response to Reviewers'.
- A marked-up copy of your manuscript that highlights changes made to the original version. You should upload this as a separate file labeled 'Revised Manuscript with Track Changes'.
- An unmarked version of your revised paper without tracked changes. You should upload this as a separate file labeled 'Manuscript'.

If you would like to make changes to your financial disclosure, please include your updated statement in your cover letter. Guidelines for resubmitting your figure files are available below the reviewer comments at the end of this letter.

If applicable, we recommend that you deposit your laboratory protocols in protocols.io to enhance the reproducibility of your results. Protocols.io assigns your protocol its own identifier (DOI) so that it can be cited independently in the future. For instructions see: https://journals.plos.org/plosone/s/submission-guidelines#loc-laboratory-protocols. Additionally, PLOS ONE offers an option for publishing peer-reviewed Lab Protocol articles, which describe protocols hosted on protocols.io. Read more information on sharing protocols at https://plos.org/protocols?utm_medium=editorial-email&utm_source=authorletters&utm_campaign=protocols.

As the corresponding author, your ORCID iD is verified in the submission system and will appear in the published article. PLOS supports the use of ORCID, and we encourage all coauthors to register for an ORCID iD and use it as well. Please encourage your coauthors to verify their ORCID iD within the submission system before final acceptance, as unverified ORCID iDs will not appear in the published article. *Only*  the individual author can complete the verification step; PLOS staff *cannot*  verify ORCID iDs on behalf of authors.

We look forward to receiving your revised manuscript.

Kind regards,

Jung Yeon Heo

Academic Editor

PLOS One

**Journal Requirements:**

1. If the reviewer comments include a recommendation to cite specific previously published works, please review and evaluate these publications to determine whether they are relevant and should be cited. There is no requirement to cite these works unless the editor has indicated otherwise.

2. Please review your reference list to ensure that it is complete and correct. If you have cited papers that have been retracted, please include the rationale for doing so in the manuscript text, or remove these references and replace them with relevant current references. Any changes to the reference list should be mentioned in the rebuttal letter that accompanies your revised manuscript. If you need to cite a retracted article, indicate the article’s retracted status in the References list and also include a citation and full reference for the retraction notice.

**Additional Editor Comments :**

The multivariable Firth penalized logistic regression should be presented more explicitly as an exploratory analysis. Although Firth’s method is appropriate for sparse data and separation, it cannot overcome the fundamental limitation imposed by only four recurrence events. Moreover, because BMI was missing in 23 patients, the adjusted model appears to have been based on a complete-case sample and potentially fewer than four recurrence events. Please clearly report how missing BMI data were handled, as well as the exact number of patients and recurrence events included in the multivariable model, preferably in a footnote to Table 4. The adjusted estimates should not be described as demonstrating independent effects or model stability. Instead, they should be framed as highly imprecise, hypothesis-generating associations. The terms “primary analysis,” “independent predictor,” and “independent determinant” should therefore be replaced with more cautious wording such as “exploratory adjusted analysis” and “association with recurrence.” The Abstract should also avoid emphasizing the extreme adjusted OR for low BMI without clearly stating its substantial imprecision.

[Note: HTML markup is below. Please do not edit.]

Reviewers' comments:

Reviewer's Responses to Questions

**Comments to the Author**

Reviewer #1: All comments have been addressed

Reviewer #2: All comments have been addressed

**********

2. Is the manuscript technically sound, and do the data support the conclusions?

Reviewer #1: Partly

Reviewer #2: Yes

**********

3. Has the statistical analysis been performed appropriately and rigorously?

Reviewer #1: Yes

Reviewer #2: Yes

**********

4. Have the authors made all data underlying the findings in their manuscript fully available?

The PLOS Data policy

Reviewer #1: Yes

Reviewer #2: Yes

**********

5. Is the manuscript presented in an intelligible fashion and written in standard English?

Reviewer #1: Yes

Reviewer #2: Yes

**********

**Reviewer #1:**  I thank the authors for their thorough and, in several places, commendably candid response to the previous round of review. The revision meaningfully improves the manuscript: the title now reflects the analytic population, the tone of the Discussion conclusion is appropriately hedged, operational definitions for comorbidities and adverse drug reactions have been added, baseline laboratory data and missing-data patterns are now reported, and useful sensitivity analyses have been supplied.

The fundamental constraint of this study ("four recurrence events") cannot be resolved by any analytic method, and the authors now reasonably frame their findings as exploratory. My remaining concerns are therefore not requests for new analyses, but requests that the reporting be made fully consistent with that exploratory framing and fully transparent about what the primary model actually estimates. Several of these should be addressed before the manuscript is suitable for publication.

Major points

1. The Abstract conclusion remains overstated and is now inconsistent with the revised Discussion. In the response letter and in the Discussion (final paragraph), the authors state that they removed language suggesting a benefit in selected patients and reframed the findings as exploratory and imprecise. However, the Abstract conclusion still reads that "our findings indicate a potential benefit of extended treatment in selected patients, warranting prospective evaluation of risk-stratified extension strategies." As the Abstract is what most readers will see, please revise it to match the hedged wording of the Discussion conclusion.

2. The extreme low-BMI estimate is presented in the Abstract without any indication of imprecision. The Abstract reports an adjusted OR of 81.92 (95% CI, 4.95–21,694.24). This estimate arises from near-complete separation (0/110 vs. 3/26) and its magnitude is not interpretable. Consistent with the caveat the authors added to the Discussion, I recommend removing the numeric estimate from the Abstract and instead stating that low BMI was associated with recurrence but that the estimate was very imprecise.

3. The analytic sample size and number of events contributing to the multivariable model must be reported. The primary multivariable model includes BMI, which is missing in 23 patients (14.5%), and no imputation is described, implying a complete-case analysis of approximately 136 patients. Because three of the four recurrence events occurred in the low-BMI group and the fourth appears to fall among the BMI-missing patients, the multivariable model may be based on as few as three events. Please state explicitly (a) how missing BMI was handled, and (b) the actual N and number of recurrence events contributing to the multivariable model, ideally in a footnote to Table 4. This information is essential for readers to judge the primary estimate.

Minor points

4. Given the numeric corrections required in this revision, I would ask the authors to independently re-verify all figures in the final version. Specific inconsistencies I noted:

- The Discussion reports baseline AFB smear positivity in the standard group as 30.2%, whereas Table 1, the Abstract, and the Results state 29.3%. Please reconcile.

- The coordinating-institution IRB number appears as "C19ONDI0458" in the Ethics Statement but as "KC19ONDI0458" elsewhere. Please use a single consistent number.

- Two-month culture positivity is reported as 5.1% in the Discussion but 5.2% in Table 2; please standardize the rounding.

5. Supplementary materials. Please confirm that the final Supporting Information file is complete and that its table numbering matches the in-text citations (S1–S7), including the recurrence-case summary and the sensitivity analyses added in this revision. The numbering of the supplementary tables and the in-text references should be checked against one another.

Overall, I consider these to be addressable revisions. Provided with appropriate minor revisions, the manuscript would represent a sound, appropriately hedged contribution of real-world data on this clinically relevant question.

**Reviewer #2:**  Dear Authors,

Thank you for your thorough and thoughtful revision. The manuscript has been substantially improved, and I appreciate the considerable effort taken to address each of my previous comments. The revised title now accurately reflects the study population, the survivorship bias and confounding by indication are explicitly discussed, comorbidity and adverse drug reaction definitions have been added, baseline laboratory data and missing data analyses are now provided, and the Discussion conclusion has been appropriately tempered. The addition of a sample size estimation for future studies and the clinical description of the four recurrence cases are also valuable.

I have only two remaining concerns, both of which should be straightforward to address.

1. Inconsistency Between the Abstract Conclusion and the Discussion Conclusion

The Discussion conclusion has been revised to state that the study was underpowered and that findings should be interpreted as exploratory, which I believe is appropriate. However, the Abstract conclusion still retains the original wording: “Cavity on chest radiograph alone may not be sufficient to justify routine treatment extension beyond 200 days after treatment success. Nevertheless, our findings indicate a potential benefit of extended treatment in selected patients.” This language is more assertive than what the Discussion now conveys and creates an internal inconsistency within the manuscript. Because the Abstract is often the only section read by many clinicians, it is important that its tone matches the more measured Discussion conclusion. I would suggest revising the Abstract conclusion to align with the revised Discussion.

2. Residual Use of “Independent Determinant” in the Discussion

In the Discussion paragraph on low BMI (line 378), the phrase “independent determinant of long-term treatment outcomes” remains. While the cautionary sentence that follows is appreciated, the term “independent determinant” still implies a level of certainty that is not supported by an estimate based on four events with a confidence interval spanning four orders of magnitude. Replacing this with more cautious language, consistent with the revisions already made elsewhere in the manuscript, would ensure internal consistency.

These are minor points that should not require substantial rewriting. Once addressed, I believe the manuscript will be suitable for publication.

Best regards.

**********

what does this mean?). If published, this will include your full peer review and any attached files.

If you choose “no”, your identity will remain anonymous but your review may still be made public.

**Do you want your identity to be public for this peer review?**  For information about this choice, including consent withdrawal, please see our Privacy Policy

Reviewer #1: No

Reviewer #2: No

**********

[NOTE: If reviewer comments were submitted as an attachment file, they will be attached to this email and accessible via the submission site. Please log into your account, locate the manuscript record, and check for the action link "View Attachments". If this link does not appear, there are no attachment files.]

To ensure your figures meet our technical requirements, please review our figure guidelines: https://journals.plos.org/plosone/s/figures

You may also use PLOS’s free figure tool, NAAS, to help you prepare publication quality figures: https://journals.plos.org/plosone/s/figures#loc-tools-for-figure-preparation.

NAAS will assess whether your figures meet our technical requirements by comparing each figure against our figure specifications.

# Author comment: Author response to Decision Letter 2

**DOI:** 10.1371/journal.pone.0357772.r004

16 Jul 2026

We sincerely thank the Editor and Reviewers for their thoughtful and constructive feedback on our manuscript. We are grateful for the opportunity to revise the manuscript further. We have carefully considered all comments and revised the manuscript accordingly to improve its clarity, consistency, and transparency. Below, we provide a point-by-point response to the Editor’s and Reviewers’ comments. All changes have been incorporated into the revised manuscript and are shown in the tracked-changes version.

Additional Editor Comments:

The multivariable Firth penalized logistic regression should be presented more explicitly as an exploratory analysis. Although Firth’s method is appropriate for sparse data and separation, it cannot overcome the fundamental limitation imposed by only four recurrence events.

Thank you for this insightful comment. We agree that the multivariable Firth penalized logistic regression should be presented more explicitly as exploratory, given the very small number of recurrence events. We revised the wording throughout the manuscript, including the Abstract conclusion, Results, and Discussion, to clarify that the adjusted analysis was exploratory and that the findings should not be interpreted as definitive evidence.

Moreover, because BMI was missing in 23 patients, the adjusted model appears to have been based on a complete-case sample and potentially fewer than four recurrence events. Please clearly report how missing BMI data were handled, as well as the exact number of patients and recurrence events included in the multivariable model, preferably in a footnote to Table 4.

We also appreciate this important point regarding the handling of missing BMI data. Because BMI data were missing in 23 patients, the multivariable model including BMI was conducted as a complete-case analysis without imputation. Accordingly, this model included 136 patients and 3 recurrence events; one recurrence case with missing BMI was excluded. To make this information clear and transparent for readers, we added a footnote to Table 4 specifying the analytic sample size and the number of recurrence events included in the multivariable model.

(Table 4, Lines 319-321)

The adjusted estimates should not be described as demonstrating independent effects or model stability. Instead, they should be framed as highly imprecise, hypothesis-generating associations. The terms “primary analysis,” “independent predictor,” and “independent determinant” should therefore be replaced with more cautious wording such as “exploratory adjusted analysis” and “association with recurrence.”

Thank you for these insightful comments. First, in the Statistical Analysis subsection of the Methods, we revised the wording in the sentences explaining why Cox proportional hazards modeling and time-dependent or continuous treatment-duration modeling were not applied. Specifically, we replaced the term “primary analysis” with “the main analytic approach” to avoid implying a definitive primary causal framework.

(‘Statistical analysis’ subsection of Method, lines 206,210)

In the Results section, we also replaced “primary analysis” with “exploratory adjusted analysis” when describing the multivariable Firth penalized logistic regression results, to more clearly reflect the exploratory nature of this analysis.

(Result section, line 307)

In addition, we revised the Discussion paragraph on low BMI by replacing phrasing that could imply low BMI was an “independent” determinant with more cautious language, stating that nutritional status may be clinically relevant when considering long-term treatment outcomes. We then linked this statement to the caution that interpretation is needed because the finding was based on very few recurrence events and had a wide confidence interval.

(Discussion section, lines 382-384)

In addition, because wording related to “stability” in the sensitivity analysis section could be misinterpreted as implying model stability, we revised the sentence to state more cautiously that analyses using alternative model specifications with varying degrees of covariate adjustment showed a similar direction of association.

(Result section, lines 328-329)

The Abstract should also avoid emphasizing the extreme adjusted OR for low BMI without clearly stating its substantial imprecision.

We also revised the Abstract to avoid emphasizing the extreme adjusted OR for low BMI. Specifically, we removed the numerical adjusted OR and confidence interval for low BMI from the Abstract and instead stated that low BMI was associated with recurrence, although the estimate was highly imprecise.

( Result of Abstract, lines 100-101)

Reviewer #1: I thank the authors for their thorough and, in several places, commendably candid response to the previous round of review. The revision meaningfully improves the manuscript: the title now reflects the analytic population, the tone of the Discussion conclusion is appropriately hedged, operational definitions for comorbidities and adverse drug reactions have been added, baseline laboratory data and missing-data patterns are now reported, and useful sensitivity analyses have been supplied.

The fundamental constraint of this study ("four recurrence events") cannot be resolved by any analytic method, and the authors now reasonably frame their findings as exploratory. My remaining concerns are therefore not requests for new analyses, but requests that the reporting be made fully consistent with that exploratory framing and fully transparent about what the primary model actually estimates. Several of these should be addressed before the manuscript is suitable for publication.

Major points

1. The Abstract conclusion remains overstated and is now inconsistent with the revised Discussion. In the response letter and in the Discussion (final paragraph), the authors state that they removed language suggesting a benefit in selected patients and reframed the findings as exploratory and imprecise. However, the Abstract conclusion still reads that "our findings indicate a potential benefit of extended treatment in selected patients, warranting prospective evaluation of risk-stratified extension strategies." As the Abstract is what most readers will see, please revise it to match the hedged wording of the Discussion conclusion.

We thank the reviewer for this important observation and agree that the Abstract conclusion was inconsistent with the revised Discussion. We have revised the Abstract conclusion to align with the hedged, exploratory framing used in the Discussion, while retaining the study's core clinical message (i.e., that cavity alone does not justify routine extension) so that the Abstract continues to convey the paper's principal contribution rather than reading as a mere restatement of its limitations. We have also revised the wording so that the reference to microbiologic response and disease burden explicitly refers to variables that future studies should systematically capture, consistent with our Limitations section noting that detailed cavitation-specific variables (cavity size, number, extent) were not recorded in the present cohort and that two-month culture data had substantial missingness.

The revised Abstract conclusion now reads:

“Cavity on chest radiograph alone may not be sufficient to justify routine treatment extension beyond 200 days after treatment success. Because only four recurrence events were observed, the adjusted association favoring extended treatment was imprecise and should be interpreted as exploratory rather than definitive. Larger prospective studies that systematically capture microbiologic response and detailed radiologic disease burden are needed to identify which patients with cavitary disease may benefit from individualized treatment extension”

2. The extreme low-BMI estimate is presented in the Abstract without any indication of imprecision. The Abstract reports an adjusted OR of 81.92 (95% CI, 4.95–21,694.24). This estimate arises from near-complete separation (0/110 vs. 3/26) and its magnitude is not interpretable. Consistent with the caveat the authors added to the Discussion, I recommend removing the numeric estimate from the Abstract and instead stating that low BMI was associated with recurrence but that the estimate was very imprecise.

Thank you for this insightful comment. We agree that presenting the numerical low-BMI estimate in the Abstract may overemphasize an unstable estimate based on very few recurrence events. Accordingly, we removed the adjusted OR and confidence interval from the Abstract and revised the sentence to state that low BMI was associated with recurrence, while emphasizing that the estimate was highly imprecise.

The revised sentence in the Abstract Results now reads:

"Among covariates, low body mass index was associated with recurrence, but this estimate was very imprecise and should be interpreted with caution."

In addition, consistent with this revision, we also modified the wording in the Discussion regarding low BMI. Specifically, we replaced phrasing that could imply low BMI was an “independent” determinant with more cautious language, stating that nutritional status may be clinically relevant when considering long-term treatment outcomes. We then linked this statement to the caution that interpretation is needed because the finding was based on very few recurrence events and had a wide confidence interval.

The revised sentence in the Discussion section now reads: “Our results are therefore concordant with existing evidence and suggest that nutritional status may be clinically relevant to long-term treatment outcomes.”

Furthermore, although the numeric estimate was retained in the Results section of the main text for completeness and transparency, we revised that sentence to explicitly flag its imprecision, so that readers encountering the estimate outside the Abstract are not misled by its magnitude. The revised sentence in the Results section now reads: "In contrast, low body mass index (BMI) (<18.5 kg/m²) was associated with recurrence in the multivariable Firth penalized logistic regression model (OR, 81.92; 95% CI, 4.95–21,694.24; p < 0.001), although this estimate was highly imprecise due to sparse data (see Table 4 footnote)."

3. The analytic sample size and number of events contributing to the multivariable model must be reported. The primary multivariable model includes BMI, which is missing in 23 patients (14.5%), and no imputation is described, implying a complete-case analysis of approximately 136 patients. Because three of the four recurrence events occurred in the low-BMI group and the fourth appears to fall among the BMI-missing patients, the multivariable model may be based on as few as three events. Please state explicitly (a) how missing BMI was handled, and (b) the actual N and number of recurrence events contributing to the multivariable model, ideally in a footnote to Table 4. This information is essential for readers to judge the primary estimate.

Thank you for this important and insightful comment. We agree that explicitly reporting the analytic sample size and the number of recurrence events contributing to the multivariable model would improve the transparency and interpretability of the results. Because BMI data were missing in 23 patients, the primary multivariable Firth penalized logistic regression model, which included BMI, was conducted as a complete-case analysis without imputation. Accordingly, this model included 136 patients and 3 recurrence events; one recurrence case with missing BMI was excluded. To make this clearer for readers, we added a footnote to Table 4 specifying the actual analytic sample size and event count used in the multivariable model. (Result section, Table 4 footnote, lines 319-321).

In addition, in Table 4, we revised confidence interval lower bounds that had been rounded to 0.00 and reported them as <0.01 to avoid misinterpretation. (Table 4)

Minor points

4. Given the numeric corrections required in this revision, I would ask the authors to independently re-verify all figures in the final version. Specific inconsistencies I noted:

- The Discussion reports baseline AFB smear positivity in the standard group as 30.2%, whereas Table 1, the Abstract, and the Results state 29.3%. Please reconcile.

Thank you for pointing out this inconsistency. We have corrected the baseline AFB smear positivity in the Discussion from 30.2% to 29.3% for the standard-treatment group, so that it is consistent with Table 1, the Abstract, and the Results section.

(Discussion section, lines 358)

- The coordinating-institution IRB number appears as "C19ONDI0458" in the Ethics Statement but as "KC19ONDI0458" elsewhere. Please use a single consistent number.

Thank you for carefully noting this inconsistency. After rechecking the coordinating-institution IRB approval number, we standardized it as “KC19ONDI0458” throughout the manuscript to ensure consistency.

(Methods section, lines 227)

- Two-month culture positivity is reported as 5.1% in the Discussion but 5.2% in Table 2; please standardize the rounding.

Thank you for carefully noting this inconsistency. We have standardized the reported proportion of two-month culture positivity as 5.2% in the Discussion, consistent with the value presented in Table 2.

(Discussion section, lines 419)

5. Supplementary materials. Please confirm that the final Supporting Information file is complete and that its table numbering matches the in-text citations (S1–S7), including the recurrence-case summary and the sensitivity analyses added in this revision. The numbering of the supplementary tables and the in-text references should be checked against one another.

Thank you for this helpful comment. We carefully reviewed the final Supporting Information file and cross-checked all supplementary table numbers against the corresponding in-text citations. We confirmed that the Supporting Information file includes Supporting Tables S1–S7, including the recurrence-case summary and the sensitivity analyses added during revision, and that the numbering of the supplementary tables is consistent with the in-text references.

Reviewer #2: Dear Authors,

Thank you for your thorough and thoughtful revision. The manuscript has been substantially improved, and I appreciate the considerable effort taken to address each of my previous comments. The revised title now accurately reflects the study population, the survivorship bias and confounding by indication are explicitly discussed, comorbidity and adverse drug reaction definitions have been added, baseline laboratory data and missing data analyses are now provided, and the Discussion conclusion has been appropriately tempered. The addition of a sample size estimation for future studies and the clinical description of the four recurrence cases are also valuable.

I have only two remaining concerns, both of which should be straightforward to address.

1. Inconsistency Between the Abstract Conclusion and the Discussion Conclusion

The Discussion conclusion has been revised to state that the study was underpowered and that findings should be interpreted as exploratory, which I believe is appropriate. However, the Abstract conclusion still retains the original wording: “Cavity on chest radiograph alone may not be sufficient to justify routine treatment extension beyond 200 days after treatment success. Nevertheless, our findings indicate a potential benefit of extended treatment in selected patients.” This language is more assertive than what the Discussion now conveys and creates an internal inconsistency within the manuscript. Because the Abstract is often the only section read by many clinicians, it is important that its tone matches the more measured Discussion conclusion. I would suggest revising the Abstract conclusion to align with the revised Discussion.

Thank you for this important and helpful comment. We fully agree that the Abstract conclusion should be consistent with the more cautious wording of the Discussion conclusion, particularly given the limited number of recurrence events. Accordingly, we have revised the Abstract conclusion to more clearly emphasize the small number of recurrence events, the imprecisi

Attachment Submitted filename: Response letter.docx

# Peer review: Decision Letter 2

**DOI:** 10.1371/journal.pone.0357772.r005

**Authors:** Jung Yeon Heo

23 Aug 2026

<p>Extended Treatment Duration and Recurrence in Successfully Treated Drug-Susceptible Pulmonary Tuberculosis with Cavity on Chest Radiograph

PONE-D-26-05397R2

Dear Dr. Kwon,

We’re pleased to inform you that your manuscript has been judged scientifically suitable for publication and will be formally accepted for publication once it meets all outstanding technical requirements.

Within one week, you’ll receive an e-mail detailing the required amendments. When these have been addressed, you’ll receive a formal acceptance letter and your manuscript will be scheduled for publication.

An invoice will be generated when your article is formally accepted. Please note, if your institution has a publishing partnership with PLOS and your article meets the relevant criteria, all or part of your publication costs will be covered. Please make sure your user information is up-to-date by logging into Editorial Manager at Editorial Manager® and clicking the ‘Update My Information' link at the top of the page. For questions related to billing, please contact billing support.

If your institution or institutions have a press office, please notify them about your upcoming paper to help maximize its impact. If they’ll be preparing press materials, please inform our press team as soon as possible -- no later than 48 hours after receiving the formal acceptance. Your manuscript will remain under strict press embargo until 2 pm Eastern Time on the date of publication. For more information, please contact onepress@plos.org.

Kind regards,

Jung Yeon Heo

Academic Editor

PLOS One

Additional Editor Comments:

IRB approval number remains inconsistent in the methods section. Please verify the correct IRB approval number and ensure that it is reported consistently throughout the manuscript.

Reviewers' comments:

Reviewer's Responses to Questions

**Comments to the Author**

Reviewer #1: All comments have been addressed

Reviewer #2: All comments have been addressed

**********

2. Is the manuscript technically sound, and do the data support the conclusions?

Reviewer #1: Yes

Reviewer #2: Yes

**********

3. Has the statistical analysis been performed appropriately and rigorously?

Reviewer #1: Yes

Reviewer #2: Yes

**********

4. Have the authors made all data underlying the findings in their manuscript fully available?

The PLOS Data policy

Reviewer #1: Yes

Reviewer #2: Yes

**********

5. Is the manuscript presented in an intelligible fashion and written in standard English?

Reviewer #1: Yes

Reviewer #2: Yes

**********

Reviewer #1: I thank the authors for their complete response. All three major points are fully addressed, and the minor numeric inconsistencies have also been reconciled. The manuscript now presents its findings honestly as exploratory, and I have no further substantive concerns.

One production-stage point, not requiring further review: the coordinating-institution IRB number reads "KC19ONDI0458" in the Methods but "C19ONDI0458" in the Ethics Statement field; please make the two consistent.

Reviewer #2: Dear authrors,

Thank you for careful and sound response to comments from the reviewer. All requested revisions have been made accurately.

The manuscript now presents a transparent and appropriately cautious analysis of an important clinical question. Despite the inherent limitation of four recurrence events, the study provides meaningful real-world evidence from a well-characterized multicenter Korean cohort and clearly frames its findings as hypothesis-generating. I have no further comments and am satisfied that the manuscript is suitable for publication.

Best regards.

**********

what does this mean?). If published, this will include your full peer review and any attached files.

If you choose “no”, your identity will remain anonymous but your review may still be made public.

**Do you want your identity to be public for this peer review?**  For information about this choice, including consent withdrawal, please see our Privacy Policy

Reviewer #1: No

Reviewer #2: No

**********

# Editor report: Acceptance letter

**DOI:** 10.1371/journal.pone.0357772.r006

**Authors:** Jung Yeon Heo

PONE-D-26-05397R2

PLOS One

Dear Dr. Kwon,

I'm pleased to inform you that your manuscript has been deemed suitable for publication in PLOS One. Congratulations! Your manuscript is now being handed over to our production team.

At this stage, our production department will prepare your paper for publication. This includes ensuring the following:

* All references, tables, and figures are properly cited

* All relevant supporting information is included in the manuscript submission,

* There are no issues that prevent the paper from being properly typeset

You will receive further instructions from the production team, including instructions on how to review your proof when it is ready. Please keep in mind that we are working through a large volume of accepted articles, so please give us a few days to review your paper and let you know the next and final steps.

Lastly, if your institution or institutions have a press office, please let them know about your upcoming paper now to help maximize its impact. If they'll be preparing press materials, please inform our press team within the next 48 hours. Your manuscript will remain under strict press embargo until 2 pm Eastern Time on the date of publication. For more information, please contact onepress@plos.org.

You will receive an invoice from PLOS for your publication fee after your manuscript has reached the completed accept phase. If you receive an email requesting payment before acceptance or for any other service, this may be a phishing scheme. Learn how to identify phishing emails and protect your accounts at https://explore.plos.org/phishing.

If we can help with anything else, please email us at customercare@plos.org.

Thank you for submitting your work to PLOS One and supporting open access.

Kind regards,

PLOS One Editorial Office Staff

on behalf of

Dr. Jung Yeon Heo

Academic Editor

PLOS One
